# The Kühtai data set: 25 years of lysimetric, snow pillow, and meteorological measurements

**DOI:** 10.1002/2017WR020445

**Published:** 2017-06-13

**Authors:** P. Krajči, R. Kirnbauer, J. Parajka, J. Schöber, G. Blöschl

**Affiliations:** ^1^ Institute of Hydrology, Slovak Academy of Sciences Liptovsky Mikulas Slovakia; ^2^ Avalanche Prevention Centre, Mountain Rescue Service Liptovský Hrádok Slovakia; ^3^ Institute for Hydraulic and Water Resources Engineering TU Wien, Vienna Austria; ^4^ TIWAG‐Tiroler Wasserkraft AG, Hydropower planning department Innsbruck Austria

**Keywords:** snow pillow, snow lysimeter, long‐term data set, Kühtai

## Abstract

Snow measurements at the Kühtai station in Tirol, Austria, (1920 m.a.s.l.) are described. The data set includes snow water equivalent from a 10 m^2^ snow pillow, snow melt outflow from a 10 m^2^ snow lysimeter placed at the same location as the pillow, meteorological data (precipitation, incoming shortwave radiation, reflected shortwave radiation, air temperature, relative air humidity, and wind speed), and other data (snow depths, snow temperatures at seven heights) from the period October 1990 to May 2015. All data have been quality checked, and gaps in the meteorological data have been filled in. The data set is unique in that all data are available at a temporal resolution of 15 min over a period of 25 years with minimal changes in the experimental setup. The data set can therefore be used to analyze snow pack processes over a long‐time period, including their extremes and long‐term changes, in an Alpine climate. Analyses may benefit from the combined measurement of snow water equivalent, lysimeter outflow, and precipitation at a wind‐sheltered alpine site. An example use of data shows the temporal variability of daily and 1 April snow water equivalent observed at the Kühtai site. The results indicate that the snow water equivalent maximum varies between 200 and more than 500 mm w.e., but there is no statistically significant temporal trend in the period 1990–2015.

## Key Points

- A 25 year snow data set in an Alpine setting with minimal changes in the experimental setup
- Meteorological and snow data at 15 min resolution were quality checked and gaps were filled
- The data set can be used for analysing long‐term snow processes and testing snow models

## 1 Introduction

Long‐term observations of processes leading to snow pack accumulation and melt are needed to better understand the processes and factors that control the variability in snow melt runoff generation, particularly during extreme events [*Blöschl*, 1999].

The objective of this paper is to present a data set of long‐term observations from an experimental snow lysimeter plot in Kühtai (Austrian Alps). The snow monitoring site was set up in 1989/1990 on the site of an existing meteorological station near Kühtai hydropower station and Längental reservoir [*Kirnbauer and Blöschl*, 1990]. The data from the site have been used in the early 1990s to test snow melt and accumulation models [*Blöschl and Kirnbauer*, 1991, 1992; *Blöschl et al*., 1991], and more recently to test snow density models [*Schöber et al*., 2016].

Previous snow model intercomparison projects [*Etchevers et al*., 2004; *Essery et al*., 2009; *Rutter et al*., 2009] stressed the importance of high‐quality hydrometeorological data sets for evaluating hydrological models, weather forecasts, climate predictions, and remote‐sensing products. The Kühtai data set provides detailed information on the amount of water stored in the snowpack, melt dynamics and corresponding meteorological, snow depth, and profile snow temperature data in the period 1990–2015. In comparison to other published snow data sets [e.g., *Marks et al*., 2001; *Reba et al*., 2011; *Morin et al*., 2012; *Landry et al*., 2014; *Wayand et al*., 2015; WSL Institute for Snow and Avalanche Research SLF, 2015; *Essery et al*., 2016], it provides meteorological and snow observations data at shorter temporal resolution (15 min). Additional interesting features about the Kühtai data set are the identical location of the snow pillow and the lysimeter, an almost unchanged setup over the past 25 years, and a wind sheltered, alpine setting.

This data set is introduced by describing the Kühtai study site in section 2. All meteorological and snow observations are presented in section 3. Quality control and methods used to fill missing data are presented in section 4. An example of the long‐term snow data are provided in section 5. Section 6 summarized the conclusions.

## 2 Kühtai Site Description

**Figure 1:**
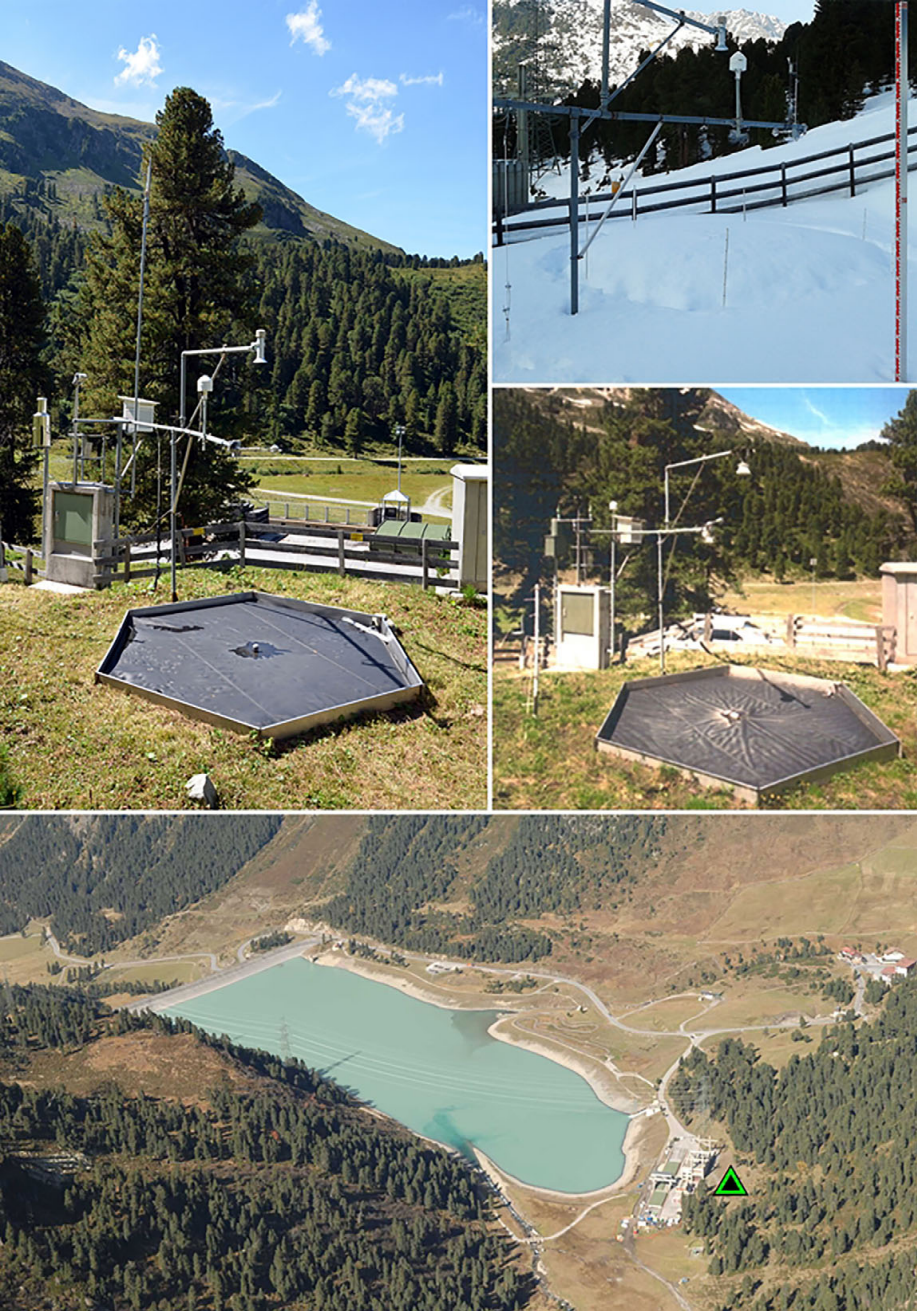
Kühtai snow monitoring station. (top left) September 2016; (top right) March 2015 when snow water equivalent was 345 mm; (middle right) September 1990. A comparison of photos shows that the station setup remained almost unchanged in the period 1990–2015. (bottom) Setting of the station. The triangle indicates location of the station.

The Kühtai snow monitoring station is located about 30 km west of Innsbruck, Tyrol (latitude 47.207111°N, longitude 11.005999°E) at an elevation of 1920 m above sea level (for location map, see *Kirnbauer and Blöschl* [1990]). It is situated in a steep alpine valley of the Austrian Alps, which limits the sun exposure during winter months. The experimental plot is surrounded by typical timberline vegetation with Alpine roses, meadows, and scattered cembra‐pines (Figure 1).

The climate of the experimental plot is characterized by a mean annual precipitation of 1200 mm/yr and a mean annual air temperature of about 2°C. The annual precipitation total is slightly higher than that of other stations in the area at the same elevation due to its rather sheltered location within the Alps. Snowpack formation typically starts in November and reaches its maximum depth of about 1.10–2.00 m in March. In late March, the snowpack tends to become isothermal, and after that it is subject to superficial nighttime refreezing. Snowmelt is most intense from late April until mid‐May, when the snow cover typically disappears.

The design of the lysimetric snow pillow is based on a device described by *Engelen et al*. [1984]. The hexagonal rubber pillow of 10 m^2^ in area, filled with a mixture of antifreeze and water, is connected to a stand pipe where water level is measured. The meltwater draining the pillow is collected in a gutter at the edge of the lysimeter and measured by a tipping bucket of 0.05 mm resolution. Lateral inflow to the lysimeter is prevented by a drainage surrounding the device and a 20 cm metal lip [*Kirnbauer and Blöschl*, 1990].

Staff from the nearby hydropower station measure snow depth and new snow height every morning, and these data are used to check automatic weather station data. The data set is unique in that the measurement set up of the key parameters, snow water equivalent (SWE), and snowpack drainage, has not been changed in the past 25 years.

## 3 Data Description

The data set provides continuous and quality controlled time series of measured meteorological and snow data in the period October 1990 to May 2015. Table 1 describes the instruments used for the measurements, their date of installation, and period of observation. Each measured characteristic is described below. The time series of the meteorological and snow data are plotted in the figures of the supporting information.

**Table 1:** Summary of Instruments at the Kühtai Snow Monitoring Station, Period of Operation, Height Above Ground of the Sensors and Indication Whether Data Gaps Have Been Filled in to Obtain a Complete Data Set for the Period 1990–2015^a^

Variable	Sensor	Period of Operation	Height (m)	Filled In
Air temperature	Kroneis NTC	1990–2015	2.6	Yes
Relative humidity	Pernix hair hygrometer	1990–2015	2.6	Yes
Incoming shortwave radiation	Schenk Starpyranometer 8101	1990–2015	3	Yes
Reflected shortwave radiation	EPPLEY PSP18985F3 Schenk double pyranometer 8104	1991–2012, 2012–2015	2.6	No
Wind speed	Kroneis cup anemometer	1991–1999	2.6	Yes
Precipitation	Self‐cast tipping bucket, OTT Pluvio	1990–2001, 2001–2015	2.8	Yes
Snow water equivalent	OTT Thalimedes float Piezometer E + H Deltapilot M	1990–2015	0	No
Snow lysimeter outflow	Snow lysimeter and tipping bucket	1991–2015	0	No
Snow depth	USH‐8 ultrasonic device, Manual observations	1990–2015	3.6 ‐	No
Snow temperature 20cm	Kroneis PT100	1991–2015	0.2	No
Snow temperature 40cm	Kroneis PT100	1991–2015	0.4	No
Snow temperature 60cm	Kroneis PT100	1991–2015	0.6	No
Snow temperature 80cm	Kroneis PT100	1991–2015	0.8	No
Snow temperature 100cm	Kroneis PT100	1991–2015	1.0	No
Snow temperature 120cm	Kroneis PT100	1991–2015	1.2	No
Snow temperature 140cm	Kroneis PT100	1991–2015	1.4	No

The automatic measurements are made at 1 min intervals, and records are stored as 15 min totals (precipitation and snow lysimeter outflow) or 15 min averages (air and snow temperatures, relative humidity, shortwave radiation, wind speed, SWE, and snow depth). Measurements from, e.g., 07:01 until 07:15 correspond to the 07:15 time stamp of the aggregated data.

### 3.1 Meteorological Measurements

#### 3.1.1 Air Temperature and Relative Humidity

Air temperature and relative humidity sensors were installed in a meteorological shelter 2.6 m above the ground surface. Snow depth, typically, is 0.6 m or higher from January to April, so the height of 2.6 m corresponds to an approximate height of 2 m above the snow surface. The installed Kroneis NTC sensor measures air temperatures in the range −50 to +50°C with an accuracy of +/–0.15°C. The accuracy of the Pernix hygrometer is +/–3% in the range of 5–100%.

#### 3.1.2 Incoming Short Wave and Reflected Shortwave Radiation

The incoming short wave radiation was measured by a Schenk star pyranometer, which is vented and heated to avoid any adverse effects of dew, frost, and snow on the measurements. The sensor is mounted 3 m above the snow pillow. Reflected shortwave radiation was measured by an Eppley pyranometer at 2.6 m height, which was replaced by a Schenk double pyranometer in 2012 (see Table 1). The values of reflected shortwave radiation are included in the data set, but were only checked for negative values, and missing values were not filled in. Incoming longwave radiation is not included in the data set. Previous modelling exercises at Kühtai parameterized the incoming longwave radiation on the basis of air temperature and water vapor (an Ångström type relationship) as well as cloudiness. Cloudiness was back‐calculated from incoming shortwave radiation during the day, and interpolated during the night [see, e.g., *Blöschl et al*., 1987; *Blöschl and Kirnbauer*, 1991].

#### 3.1.3 Wind Speed

A heated device, which measured wind speed and direction in the period 1991–1999, was mounted at a height of 2.6 m. Due to its location on the eastern side of a north‐south valley, the site is completely sheltered from eastern winds. During south winds, the recorded wind speed remained low with an observed maximum speed of 4 m/s. The dominating wind direction is north‐west, also with rather low maximum speeds of 8 m/s.

#### 3.1.4 Precipitation

Unshielded precipitation data were measured using a heated self‐cast tipping bucket until 2001 and a heated OTT Pluvio device afterward. The orifice height of the instruments was installed at 2.8 m above the ground. If air temperature drops below 4°C, the OTT Pluvio device emits heat impulses (no constant warming), ensuring that measurements are not disturbed by snow and ice. The frequency of the heat impulses was adjusted carefully to avoid evaporation from the device. Measurement accuracies of both devices are on the order of 0.1 mm for precipitation totals and 0.1 mm/h for intensities.

### 3.2 Snow Measurements

#### 3.2.1 Continuous Measurements of SWE and Snow Melt From the Lysimetric Snow Pillow

Water equivalent and snow melt outflow were measured by a 10 m^2^ lysimetric snow pillow. The large size is beneficial since snow pillows are known to underestimate SWE if they are small, especially if ice lenses form within the pack due to melt‐refreeze cycles. After major snow fall events, staff from the hydropower station cut the snow around the pillow at a distance of 1 m to avoid bridging and any lateral inflows through the snow pack. When the snow pack is very shallow, at the beginning and the end of the snow season, snow melt on the pillow may be higher than on the surrounding ground due to its black color which will affect SWE [*Johnson and Marks*, 2004]. No corrections were applied for this potential effect. Snow melt outflow was measured by a tipping bucket device.

The control measurements of snow density and SWE (not shown here) demonstrated the accuracy of the snow pillow measurements. From March 2014 until April 2016, six snow pit measurements were made next to the snow pillow. A comparison between manual and automatic SWE measurements gives an R^2^ = 0.97 and an RMSE = 28 mm. The snow pit measurements were made in March, April, or May, which is the period of maximum snow pack (typically, 250–450 mm of SWE) indicating a relative RMSE of 3–10%.

#### 3.2.2 Snow Depth

Snow depth was measured at two locations. Manual observations of snow depth and new snow depth have been carried out daily at 7:00 since 1978. Automatic observations were performed by an ultrasonic device mounted 3.6 m above the snow pillow. The ultrasonic signal was automatically corrected for temperature effects on the sound velocity using an integrated air temperature sensor. However, raw snow depth measurements still fluctuated especially during radiation days. The manual and automatic measurements were typically consistent within 5 cm.

#### 3.2.3 Profile Snow Temperature

Snow temperatures were measured at 7 heights (20, 40, 60, 80, 100, 120, 140 cm) above the ground using PT 100 sensors. The sensors were mounted on a white rope, but were not shielded from solar radiation. The data are available from January 1991.

## 4 Quality Control

**Figure 2:**
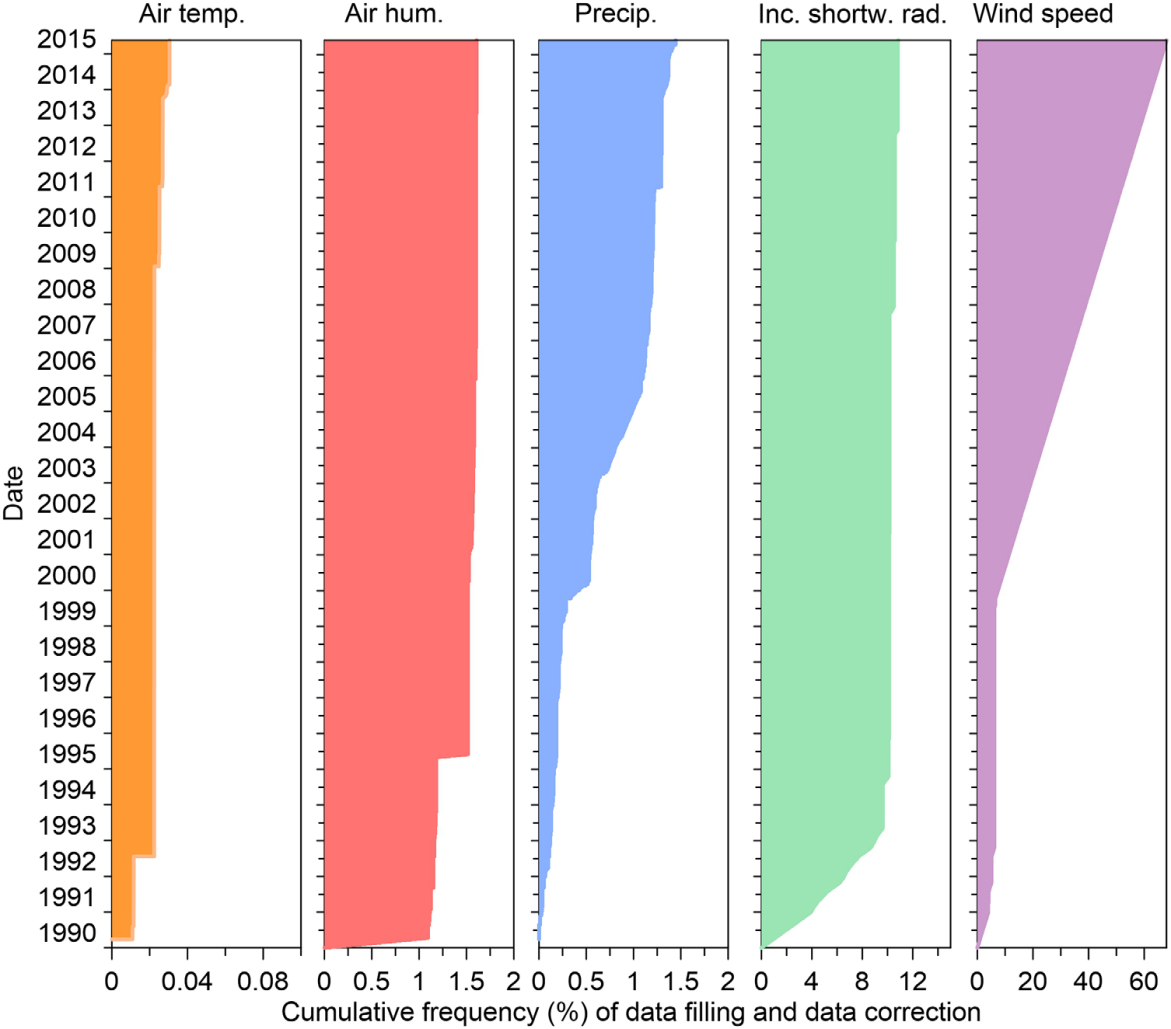
Cumulative frequency (%) of data filling and data correction for air temperature, air humidity, precipitation, incoming shortwave radiation, and wind speed expressed as the number of filled in/corrected time steps from 1 January 1990 relative to the total number of time steps.

All observed meteorological and snow data were quality controlled for unrealistic outliers, constant values, and extreme jumps. All values were carefully inspected for erroneous data by visually examining each data record. The gaps in the meteorological data were filled to allow application of snow models that account for mass balance. Figure 2 shows the cumulative frequency of data filling and correction for the meteorological data. Missing snow measurements were not filled in (except small gaps of SWE), as snow data are usually used for testing the models, rather than as an input. All corrected and filled in data are marked by flags, which are described in more detail in the following subsections. Table 2 gives the stations that were used for data filling and correction.

**Table 2:** Summary of Stations Used for Correcting and Filling in Missing Values in the Data Set^a^

Station	Latitude (°N)	Longitude (°E)	Elevation (m a.s.l.)	Variable	Distance to Kühtai (km)
Hoarlach Alm	47.15772	11.01253	1920	Air temperature, precipitation	5.5
Innsbruck University	47.26056	11.38500	578	Air humidity, wind speed, incoming shortwave radiation	29.2
Patscherkofel	47.21667	11.45000	2247	Air humidity	34.6
St. Sigmund im Sellrain	47.20332	11.10494	1520	Precipitation	7.5
Innsbruck airport	47.25889	11.35528	579	Wind speed	27.1
Oberbergbach	47.08978	11.17604	1999	Incoming shortwave radiation	18.3
Jenbach	47.38333	11.75000	530	Incoming shortwave radiation	60.3

a Geographical coordinates (latitude, longitude) are in WGS84 coordinate system.

### 4.1 Air Temperature and Relative Humidity

The air temperature measurements were checked against the Hoarlach Alm station with very good consistency. There were only 11 short data gaps of less than 1 h, which were filled by a linear interpolation of the observed data at Kühtai (flag 11). On 3 days, the gaps were longer, so data were filled by a linear regression with the Hoarlach Alm data (flag 12). The linear regression was fitted to a time window of two weeks before and after the gap with R^2^ ranging from 0.86 to 0.96.

Relative humidity was corrected and filled for less than 1.5% of the time steps. First, extreme jumps, i.e., a more than 30% change in relative humidity in 15 min and very low values (i.e., less than 10%) were identified and removed. Missing values in gaps less than 1 h were corrected by linear interpolation (flag 13). Longer gaps were filled by linear regression with data from Innsbruck University (flag 14) and, if missing, from Patscherkofel (flag 15) using the same time window as for air temperature.

### 4.2 Incoming Shortwave Radiation

To check incoming shortwave radiation, the time of sun set and sun rise was calculated [*Bivand and Lewin‐Koh*, 2016]. All night values were set to zero (flag 21). Reflected radiation measurements were used to estimate albedo. If albedo values were larger than 1.0, the incoming shortwave radiation was set to reflected radiation (flag 22). Such situations likely correspond to snow or frost covering the radiation sensor [*Morin et al*., 2012]. Missing values for less than 1 h were linearly interpolated (flag 23) from observed data. Longer gaps were filled by a linear regression with Oberbergbach (flag 24) or, if not available, with Innsbruck university (flag 25) or Jenbach (flag 26), although the latter two are valley stations that may be affected by occasional fog. The linear regression was estimated from all time steps stratified by hour of the day and month of the year. Values of incoming shortwave radiation close to the solar constant (around 1400 W.m^−2^) were not considered erroneous, as they may be affected by reflectance from nearby snow covered slopes.

### 4.3 Wind Speed

Wind speed at Kühtai was measured until the end of 1999. Missing values were filled by percentile matching with the measurements at Innsbruck University (flag 31) and Innsbruck airport (flag 32). In 2008 and 2009, additional wind speed measurements were made close to the Kühtai snow pillow at 10 m height. A few missing values in 2008 and 2009 were thus estimated by logarithmic rescaling of these measurements (flag 33).

### 4.4 Precipitation

Precipitation gaps were identified by double mass curves and filled with measurements at Hoarlach Alm and St. Sigmund im Sellrain. If the measurements at both stations were zero, the missing values at Kühtai were set to zero (flag 41). Gaps in precipitation measurements with relative humidity below 50% (flag 42) and with no change in snow depth during air temperatures below 0°C (flag (43) were set to zero as well. The rest of the missing values was filled by using a delta change procedure (flags 44 and 45). This procedure rescaled the reference observations from Horlach Alm (flag 44) or St. Sigmund im Sellrain (flag 45) by a ratio estimated from the sum of observed precipitation at Kühtai and the reference station 2 weeks before and after the gap. Finally, precipitation was checked against the increase of snow water equivalent at the snow pillow. If the daily increase in SWE was larger than 5 mm and the difference between daily precipitation and SWE increase was larger than 60% of SWE, the precipitation values (for time steps with observed precipitation) were corrected by this difference (flag 46). If the precipitation was larger than 8 mm/15 min, then the difference between SWE increase and daily precipitation sum was also redistributed to the next two 15 min time steps.

### 4.5 Snow Measurements

#### 4.5.1 Continuous Measurement of SWE and Snow Melt From the Lysimetric Snow Pillow

SWE values smaller than the resolution of the snow pillow measurements (i.e., 2 mm) were set to zero (flag 51). Gaps less than 1 day were filled by linear interpolation (flag 52). The continuous measurements of SWE were also compared to automatic snow depth measurements, and SWE values for time steps with zero snow depth were set to zero (flag 53). A few days with artificial increases in SWE without precipitation and snow depth changes were smoothed (flag 54) by removing the SWE values and interpolating them from the previous and the following days.

**Figure 3:**
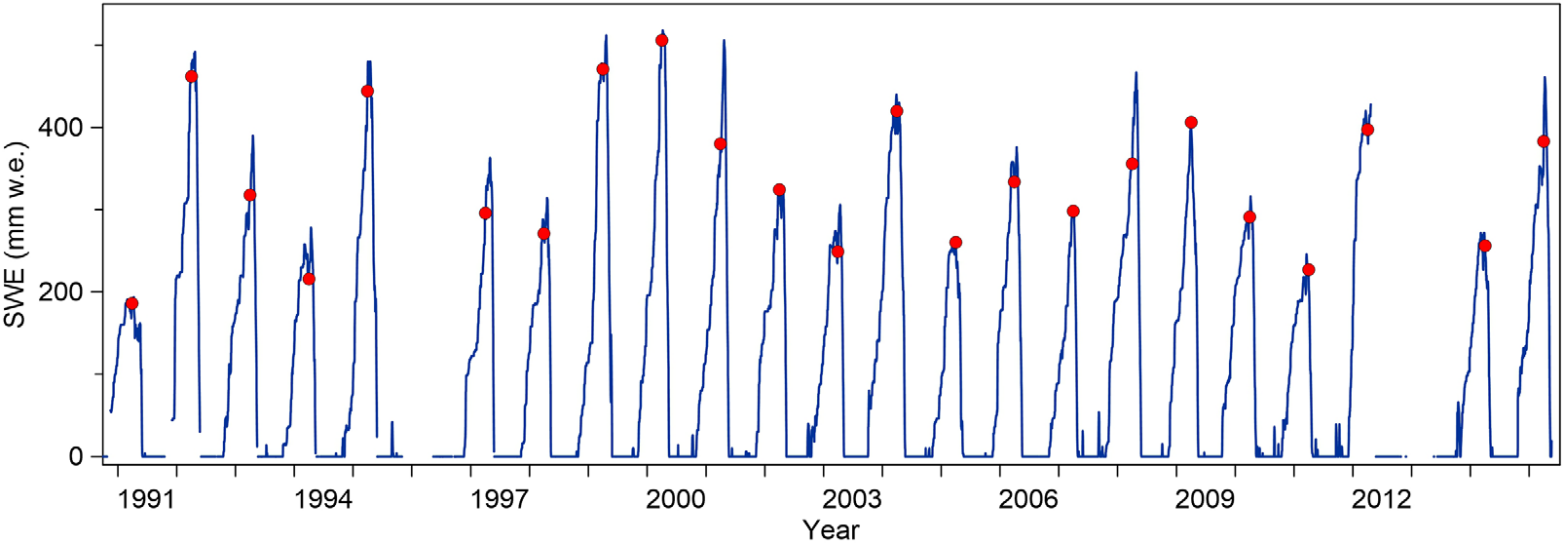
Daily snow water equivalent (SWE) at 7:00 (blue line) and SWE on 1 April (red circles) observed at the Kühtai station in the period 1990–2015.

Mass‐balance computations indicate consistency of SWE and lysimeter outflow under most conditions. Lysimeter values with more than 50% difference to the mass balance were removed (flag 55). Due to technical problems, snow pillow measurements in 1996, spring 2012, and 2013 were not available (Figure 3).

#### 4.5.2 Snow Depth

Automatic snow depth measurements were corrected for artificial jumps larger than 50 cm in 15 min (flag 61). Such values were removed and interpolated. Finally, the snow depth values were filtered by using a 50 time step averaging filter. If the difference between original and filtered values was larger than 3 cm, these values were flagged (flag 62).

#### 4.5.3 Profile Snow Temperature

Profile snow temperatures were measured by unshielded thermometers. If the snow depth was below the height of the sensor, the values were therefore removed (flag 71). Values larger than zero (flag 72) were also removed. The missing profile snow temperature values were not filled.

## 5 Example Application of Data

Long‐term observations of snow characteristics provide important information for assessing the impacts of a changing climate on available and future water resources. Figure 3 shows daily SWE observed at the Kühtai site, with the 1 April readings indicated by a circle. In many regions, 1 April values are used as consistent indicators for the maximum seasonal snow storage [*Bohr and Aguado*, 2001; *Montoya et al*., 2014]. Figure 3 indicates that the SWE maxima vary between 200 and more than 500 mm w.e. A trend analysis of the 1 April readings (not shown here) suggests that there is no statistically significant temporal trend in the period 1990–2015 (5% significance level).

*Marty et al*. [2017] report a tendency for decreasing 1 April SWE over the last six decades in the European Alps, including a sharp drop at the end of the 1980s, but no clear trend afterward. Long‐term manual snow depth data at Kühtai which have been collected from 1978 (not shown here) confirm this finding, and the lack of trend in the past 30 years from their study is also consistent with Figure 3. However, such regional comparisons need to be treated with care, as local effects may be important. The snow pillow data from Kühtai allows for detecting trends on shorter time scales, such as intra‐annual shifts of the peak SWE. Figure 3 indicates that, from 1990 to 2003, peak SWE normally occurred later than 1 April, but in the last years (2004–2014) peak SWE often occurred in March, which does point to changes in the climate at this location.

## 6 Conclusions

We present a long‐term data set of snow and meteorological measurements from an experimental snow lysimeter plot in Kühtai. The data set is unique in that all data are available at a temporal resolution of 15 min over a period of 25 years with almost no changes in the experimental setup. The data set can hence be used to analyze the long‐term dynamics of snow pack processes, including hypotheses on changes and variability of extremes, and testing of snow pack models. Data that are essential for running a snow accumulation and melt model (air temperature, relative humidity, incoming shortwave radiation, wind speed, precipitation) have been filled in to obtain complete time series. Other data that are mainly used for checking such models (reflected shortwave radiation, snow water equivalent, snow lysimeter outflow, snow depth, snow temperatures) have not been filled in. Incoming and outgoing longwave radiation, as well as the state of precipitation, are not included in the data set, and need to be modeled. All data have been quality checked and the type of each correction has been flagged.

The data set is available from the Zenodo repository doi:10.5281/zenodo.556110. The measurements are currently continued, and the data set in the repository is planned to be updated in the future.

## Supporting information

Supporting Information S1

Data Set
