# Supplementary material for: The Kühtai data set: 25 years of lysimetric, snow pillow, and meteorological measurements
Source: Water Resour Res. 2017 Jun 13;53(6):5158–65. doi: 10.1002/2017WR020445 (PMC5575548; doi:10.1002/2017WR020445)
Supplement: Supplementary file 1 — Supporting Information S1 [file WRCR-53-5158-s001.pdf]

Water Resources Research

Supporting Information for

The Kühtai data set: 25 years of lysimetric, snow pillow and meteorological measurements

P. Krajči[1,4], R. Kirnbauer[2], J. Parajka[2], J. Schöber[3], G. Blöschl[2]

[1]Institute of Hydrology, Slovak Academy of Sciences, Liptovsky Mikulas, Slovakia.

[2]Institute for Hydraulic and Water Resources Engineering, TU Wien, Vienna, Austria.

[3]TIWAG-Tiroler Wasserkraft AG, Hydropower planning department, Innsbruck, Austria.

[4]Avalanche Prevention Centre, Mountain Rescue Service, Liptovský Hrádok, Slovakia

Contents of this file

Description of dataset file
Figures S1 to S25
Table S1

Additional Supporting Information (Files uploaded separately)

The Kühtai data set S1 (snow_and_meteo_data_kuhtai_1990-2015.zip)

Introduction

The supplement contains following material:

- Description of dataset file S1 containing the meteorological data, the snow data and flags
- Table S1 indicating the type of the correction for each flag in dataset file
- Figures S1-S25 showing plots of selected meteorological and snow characteristics for individual snow seasons in the period 1990-2015.

Description of dataset file DS01.

The name of dataset file is snowdata_kuhtai_1990-2015.txt. The format of this file includes a header indicating the variable names (columns) followed by the data values (in 15 minute time steps). The delimiter is a semicolon. NA indicates missing values.

The column names represent:

1) DATE=Date in format DD.MM.YEAR
2) TIME=Time in format HH:MM (Central European Time)
3) AIRT= air temperature (oC)
4) RH= relative air humidity (%)
5) GLO= incoming short wave radiation (W.m-2)
6) WS= wind speed (m.s-1)
7) PREC= precipitation (mm/15min.)
8) SWE= snow water equivalent (mm) from snow pillow
9) LYSIM= lysimeter melt outflow (mm/15min.)
10) SND=snow depth (cm) measured by an ultrasonic device
11) SNDM=manually observed snow depth (cm) at 7:00
12) REFL=reflected shortwave radiation (W.m-2)
13) PST20= profile snow temperature at 20 cm height from the ground
14) PST40= profile snow temperature at 40 cm height from the ground
15) PST60= profile snow temperature at 60 cm height from the ground
16) PST80= profile snow temperature at 80 cm height from the ground
17) PST100= profile snow temperature at 100 cm height from the ground
18) PST120= profile snow temperature at 120 cm height from the ground
19) PST140= profile snow temperature at 140 cm height from the ground
20) F_AIRT=flag values for air temperature correction
21) F_RH=flag values for relative air humidity correction
22) F_GLO=flag values for incoming solar radiation correction
23) F_WS=flag values for wind speed correction
24) F_PREC=flag values for precipitation correction
25) F_SWE=flag values for snow water equivalent correction
26) F_LYSIM=flag values for snow lysimeter outflow correction
27) F_SND=flag values for snow depth correction
28) F_REFL=flag values for reflected shortwave radiation
29) F_PST20=flag for removed profile snow temperature at 20cm height from ground
30) F_PST40=flag for removed profile snow temperature at 40cm height from ground
31) F_PST60=flag for removed profile snow temperature at 60cm height from ground
32) F_PST80=flag for removed profile snow temperature at 80cm height from ground
33) F_PST100=flag for removed profile snow temperature at 100cm height from ground
34) F_PST120=flag for removed profile snow temperature at 120cm height from ground
35) F_PST140=flag for removed profile snow temperature at 140cm height from ground

Table S1.

Table S1 describes the corrections applied to meteorological and snow data in Kuhtai. The flag values are used in dataset file S1.

Table S1. List of flag values and corresponding corrections.

Variable	Flag value	Description
Air temperature	11	Filling shorter gaps (less than 1 hour) by linear interpolation
Air temperature	12	Filling longer gaps by linear regression with data from Hoarlach Alm
Relative air humidity	13	Filling shorter gaps (less than 1 hour) by linear interpolation
Relative air humidity	14	Filling longer gaps by linear regression with data from Innsbruck University
Relative air humidity	15	Filling longer gaps by linear regression with data from Patscherkofel
Incoming short wave radiation, reflected short wave radiation	21	Night values set to zero
Incoming short wave radiation	22	If albedo>1 incoming short wave radiation replaced by reflected short wave radiation
Incoming short wave radiation	23	Filling shorter gaps (less than 1 hour) by linear interpolation
Incoming short wave radiation	24	Filling longer gaps by linear regression with data from Oberbergbach
Incoming short wave radiation	25	Filling longer gaps by linear regression with data from Innsbruck University
Incoming short wave radiation	26	Filling longer gaps by linear regression with data from Jenbach
Reflected short wave radiation	27	Negative values are replaced by NA
Wind speed	31	Filling gaps by percentile matching with data from Innsbruck University
Wind speed	32	Filling gaps by percentile matching with data from Innsbruck Airport
Wind speed	33	Filling gaps by logarithmic rescaling of measurements at 10m height at Kühtai
Precipitation	41	Filling gaps with zero if precipitation zero at Hoarlach Alm and St. Sigmund im Sellrain
Precipitation	42	Filling gaps with zero if relative air humidity <50%
Precipitation	43	Filling gaps with zero if no change in snow depth and air temperature<0°C

Precipitation	44	Filling longer gaps by delta change procedure with data from Hoarlach Alm
Precipitation	45	Filling longer gaps by delta change procedure with data from St. Sigmund im Sellrain
Precipitation	46	Correcting and rescaling the precipitation difference to SWE, if daily increase in SWE>5 mm and the difference between daily precipitation and SWE was larger than 60%
Snow water equivalent	51	Correcting of SWE <2mm to zero
Snow water equivalent	52	Filling shorter gaps (less than 1 hour) by linear interpolation
Snow water equivalent	53	Filling and correcting SWE to zero if snow depth was zero
Snow water equivalent	54	Correcting (smoothing) diurnal SWE increase, if precipitation and snow depth change are zero
Lysimeter	55	Removing values if mass balance difference >50%
Snow depth	61	Removing artificial jumps larger than 50 cm
Snow depth	62	Flagging difference between original and filtered snow depth
Profile snow temperature (20,40,60,80,100,120,140 cm)	71	Removing values if snow depth below the height of sensor
Profile snow temperature (20,40,60,80,100,120,140 cm)	72	Removing values if profile snow temperature>0°C

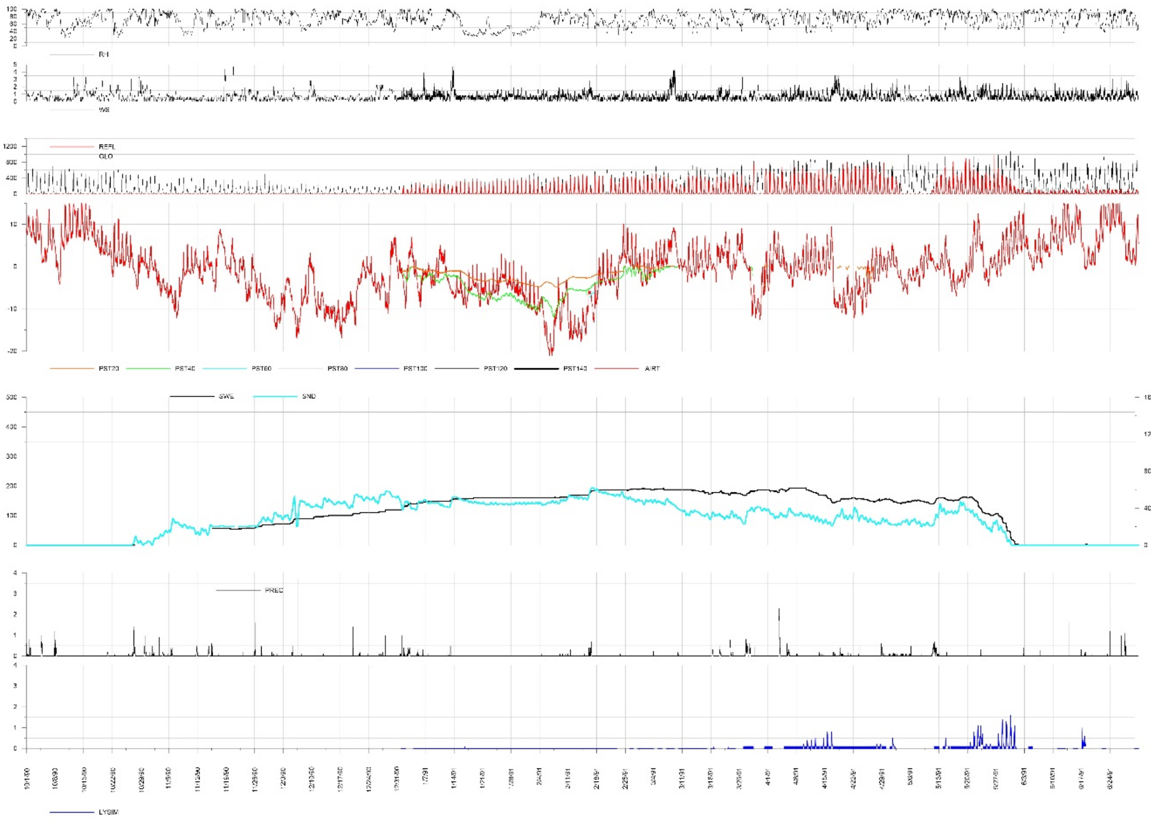

Figure S1. Plots of selected meteorological and snow characteristics in the period October 1990-June 1991.

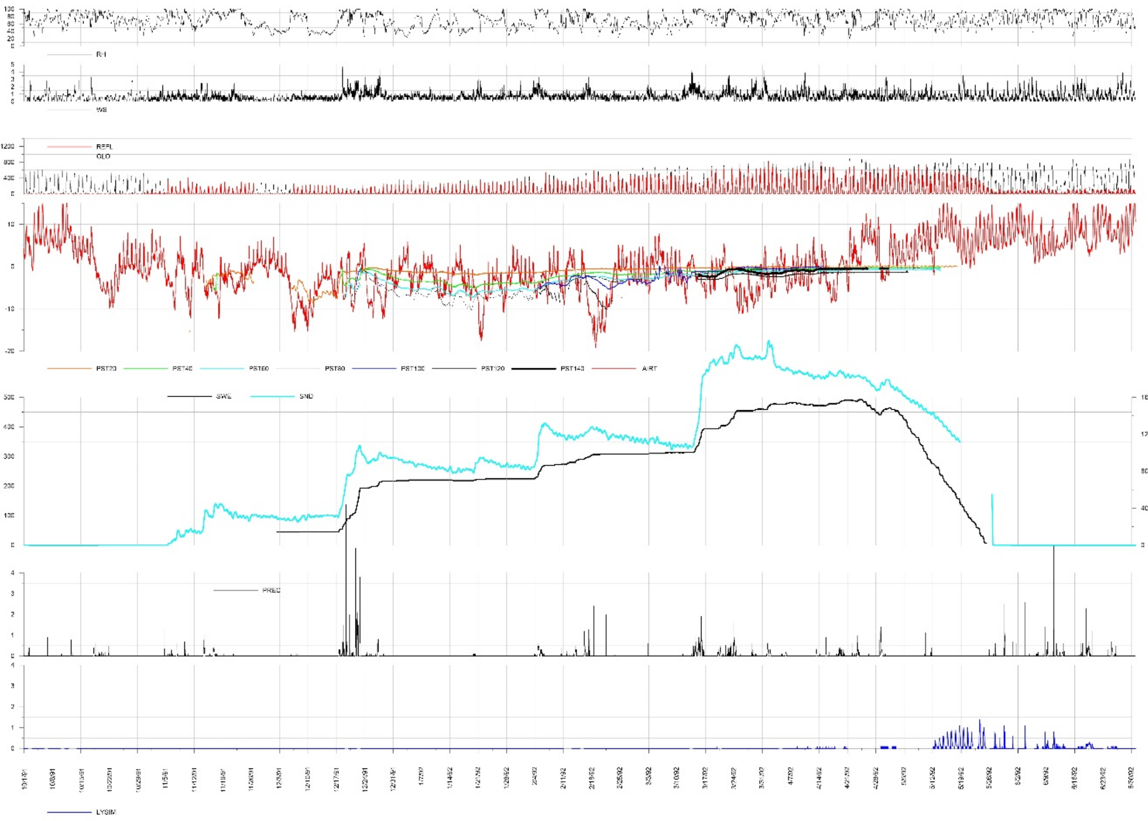

Figure S2. Plots of selected meteorological and snow characteristics in the period October 1991-June 1992.

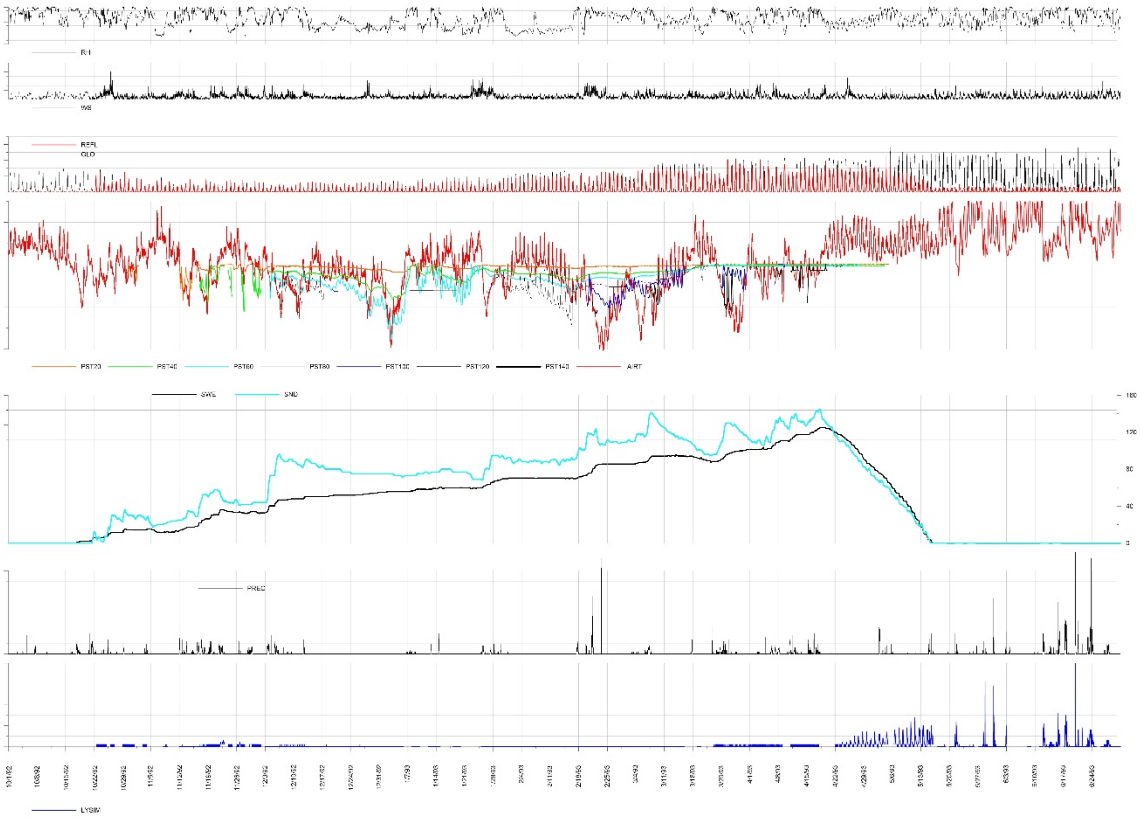

Figure S3. Plots of selected meteorological and snow characteristics in the period October 1992-June 1993.

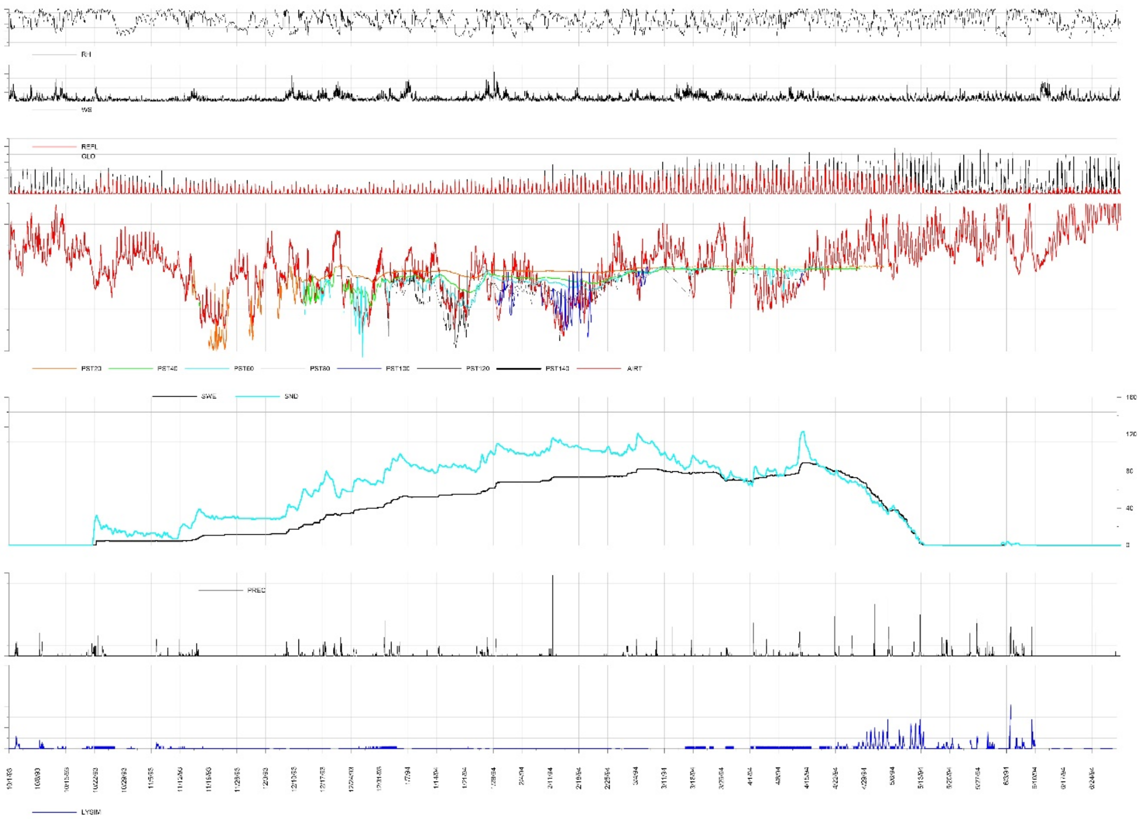

Figure S4. Plots of selected meteorological and snow characteristics in the period October 1993-June 1994.

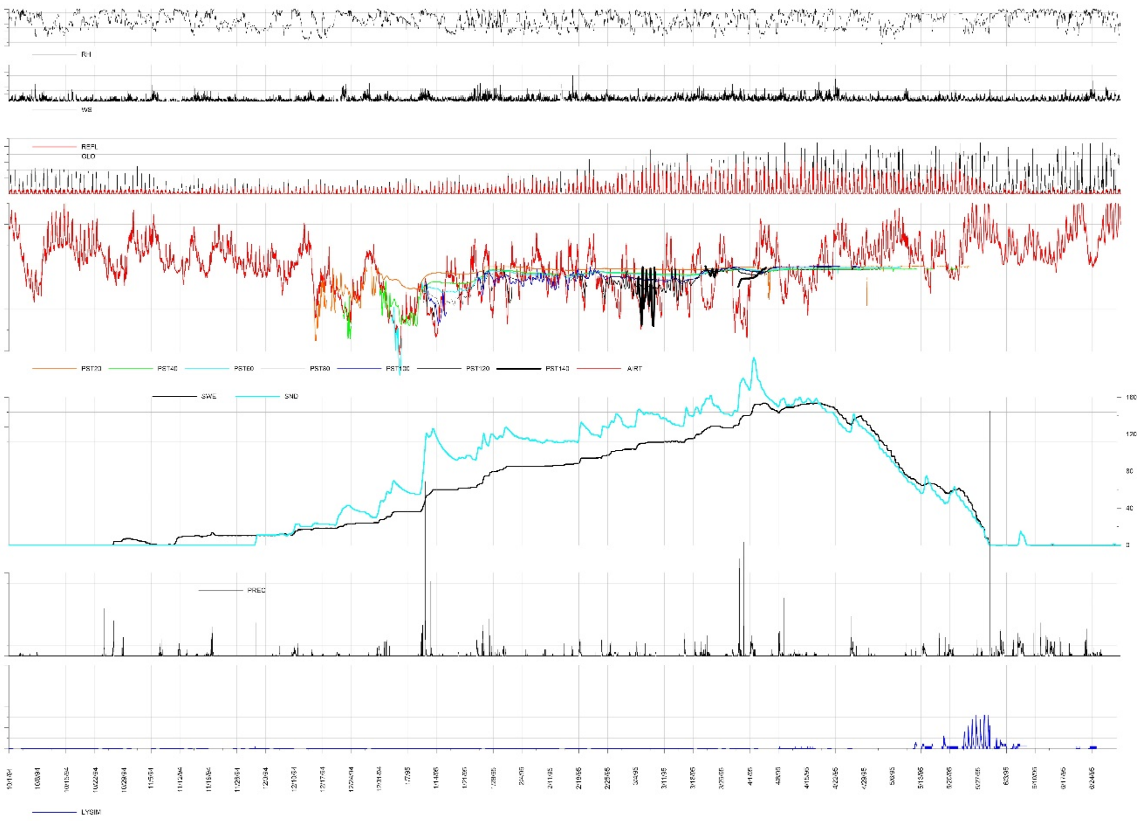

Figure S5. Plots of selected meteorological and snow characteristics in the period October 1994-June 1995.

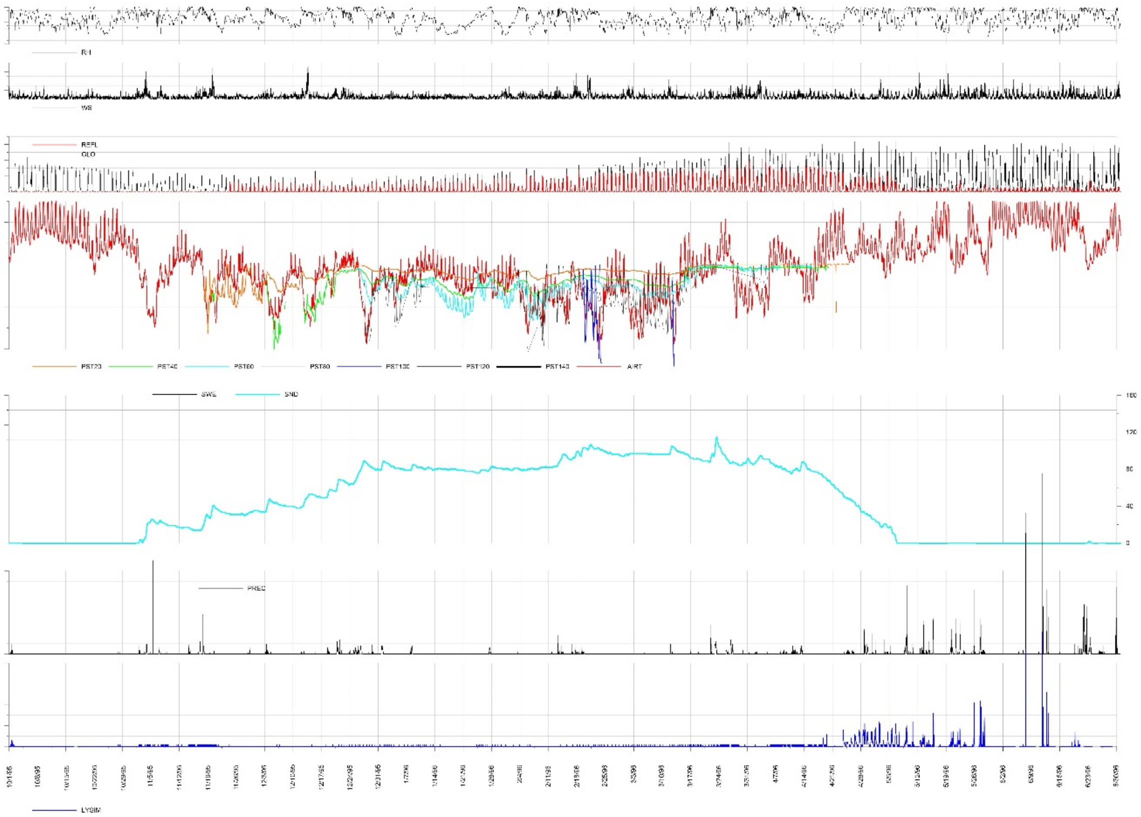

Figure S6. Plots of selected meteorological and snow characteristics in the period October 1995-June 1996.

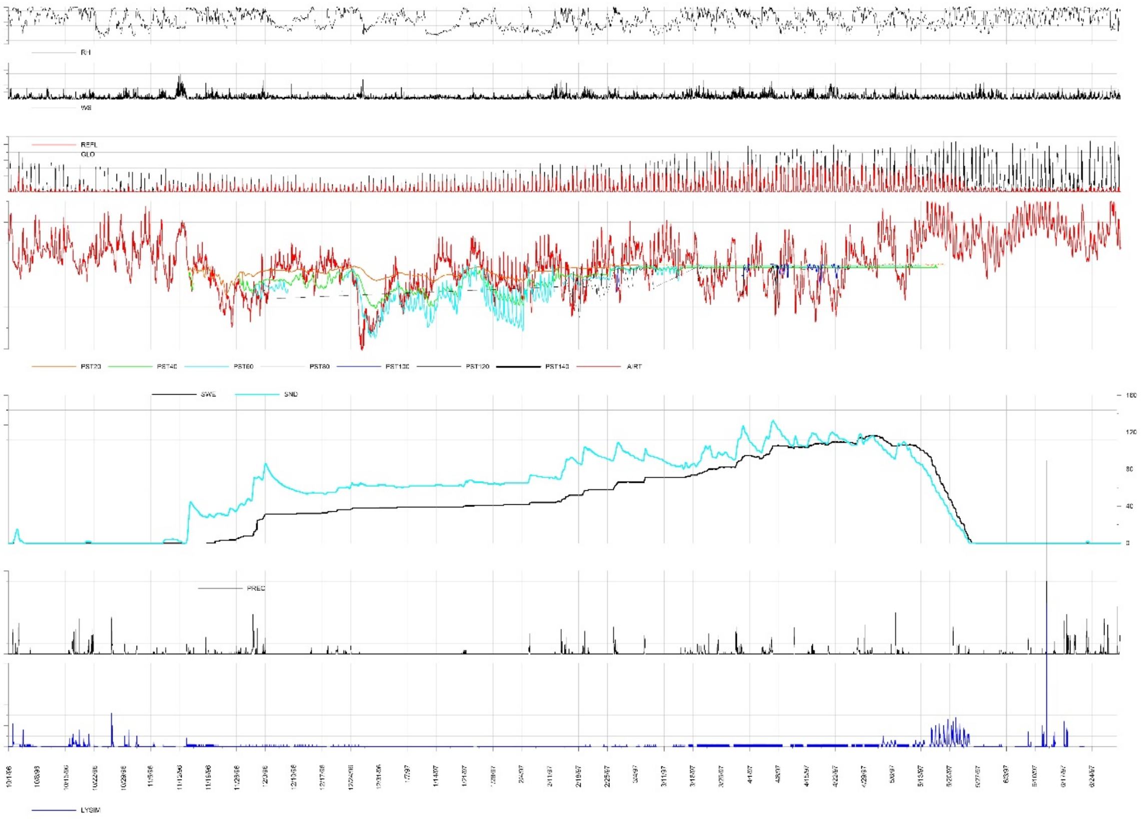

Figure S7. Plots of selected meteorological and snow characteristics in the period October 1996-June 1997.

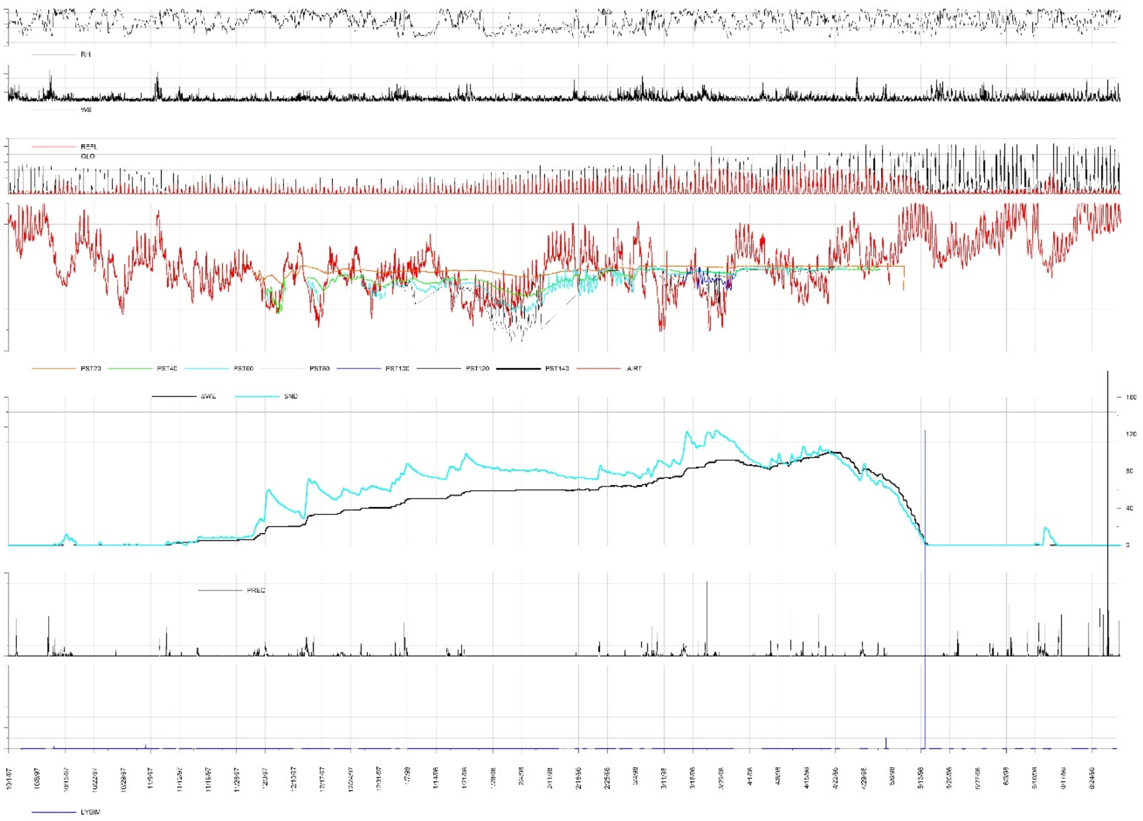

Figure S8. Plots of selected meteorological and snow characteristics in the period October 1997-June 1998.

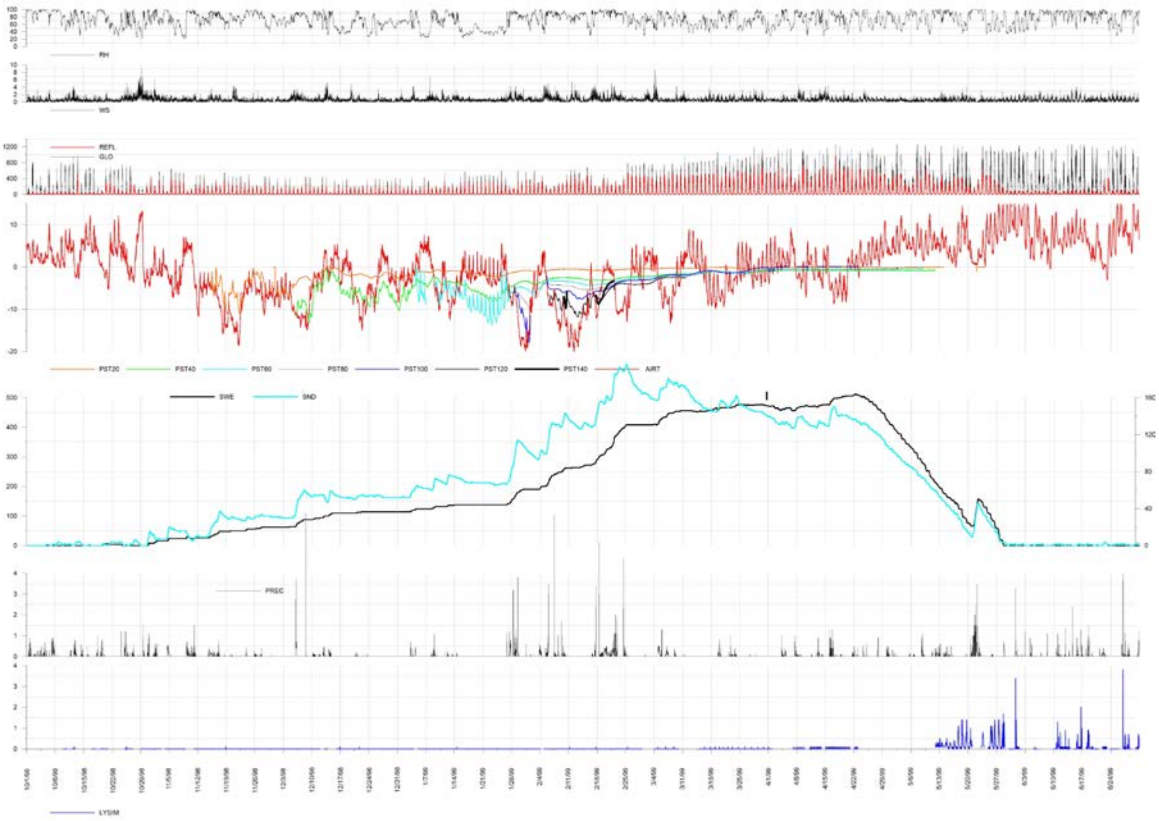

Figure S9. Plots of selected meteorological and snow characteristics in the period October 1998-June 1999.

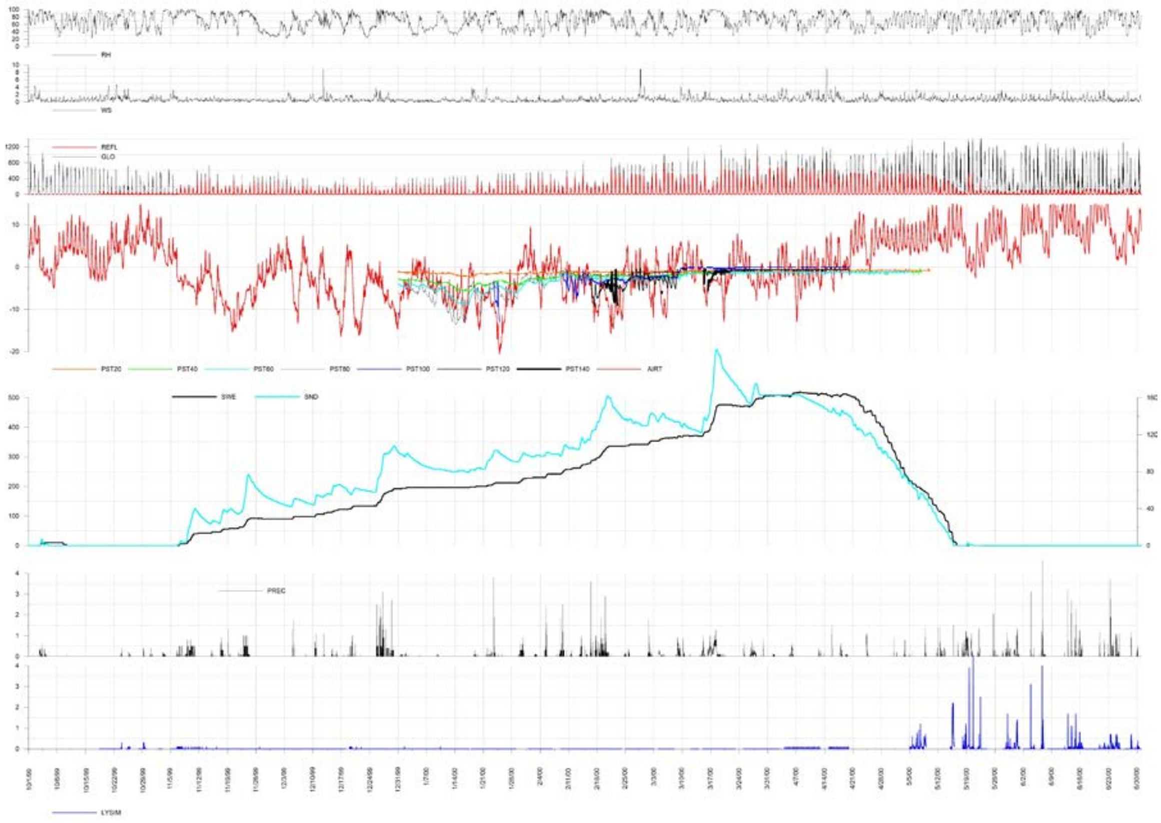

Figure S10. Plots of selected meteorological and snow characteristics in the period October 1999-June 2000.

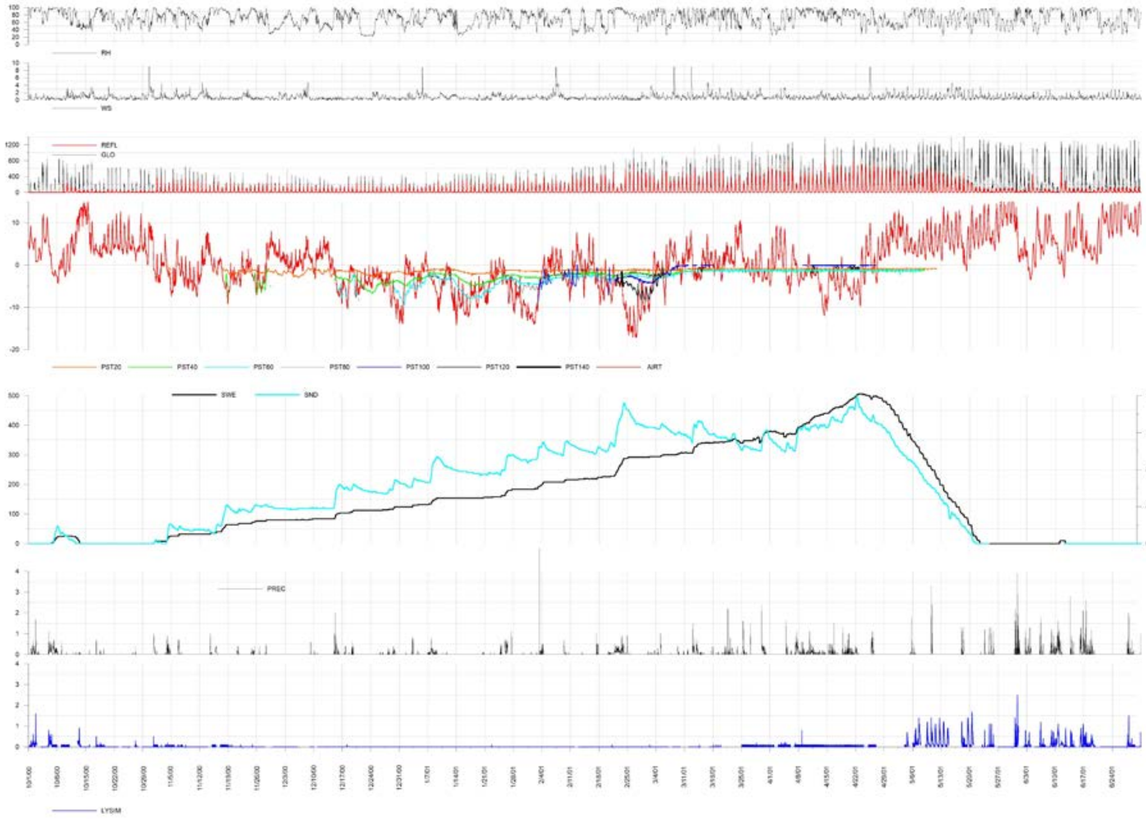

Figure S11. Plots of selected meteorological and snow characteristics in the period October 2000-June 2001.

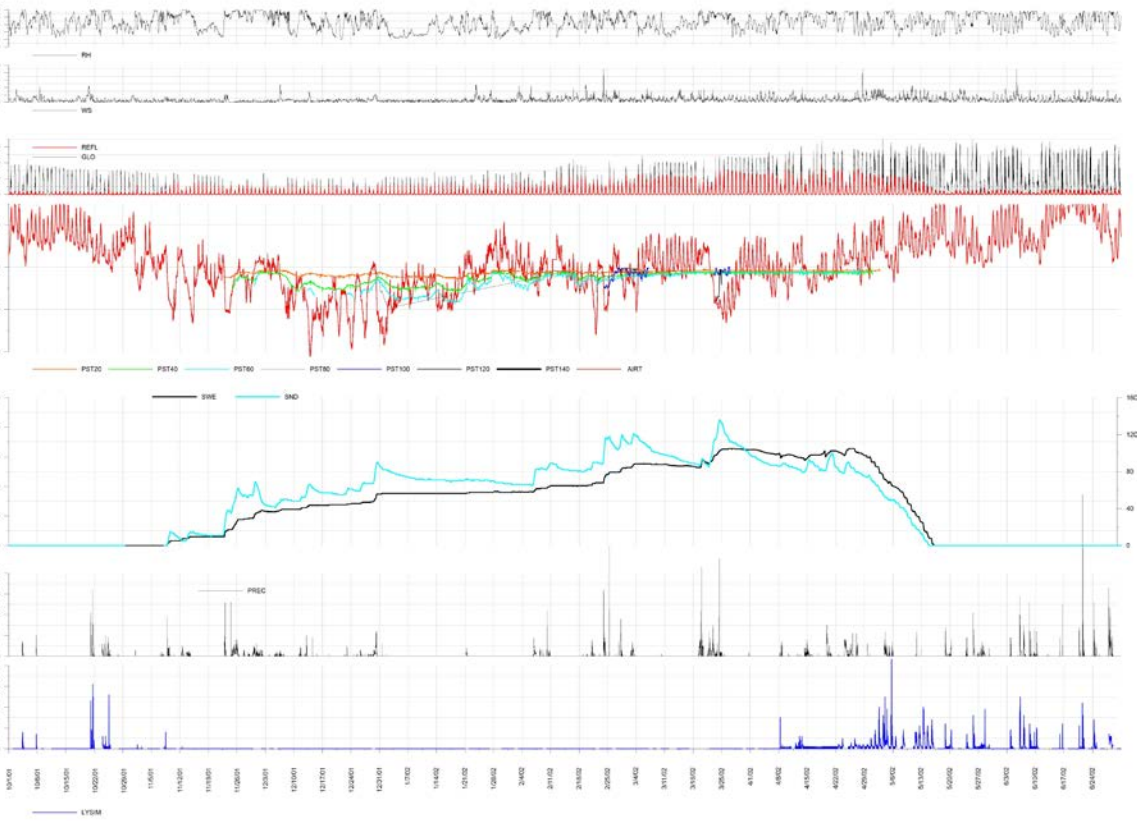

Figure S12. Plots of selected meteorological and snow characteristics in the period October 2001-June 2002.

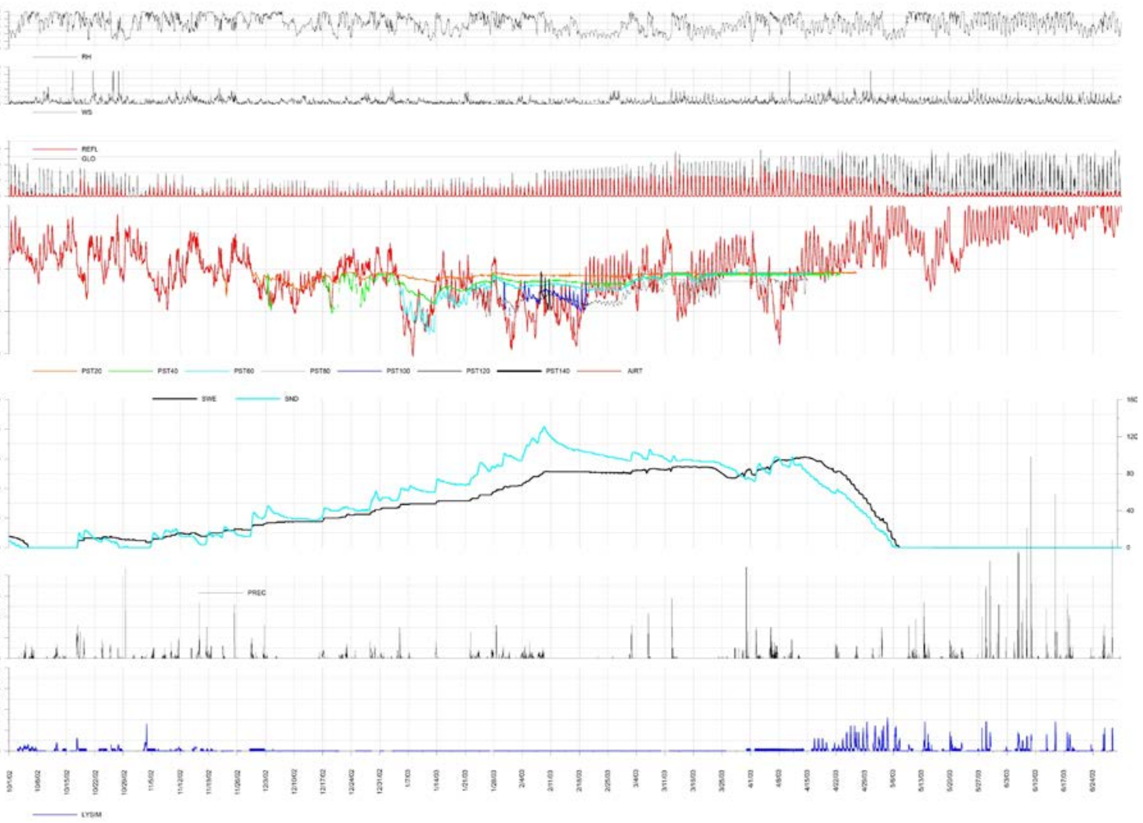

Figure S13. Plots of selected meteorological and snow characteristics in the period October 2002-June 2003.

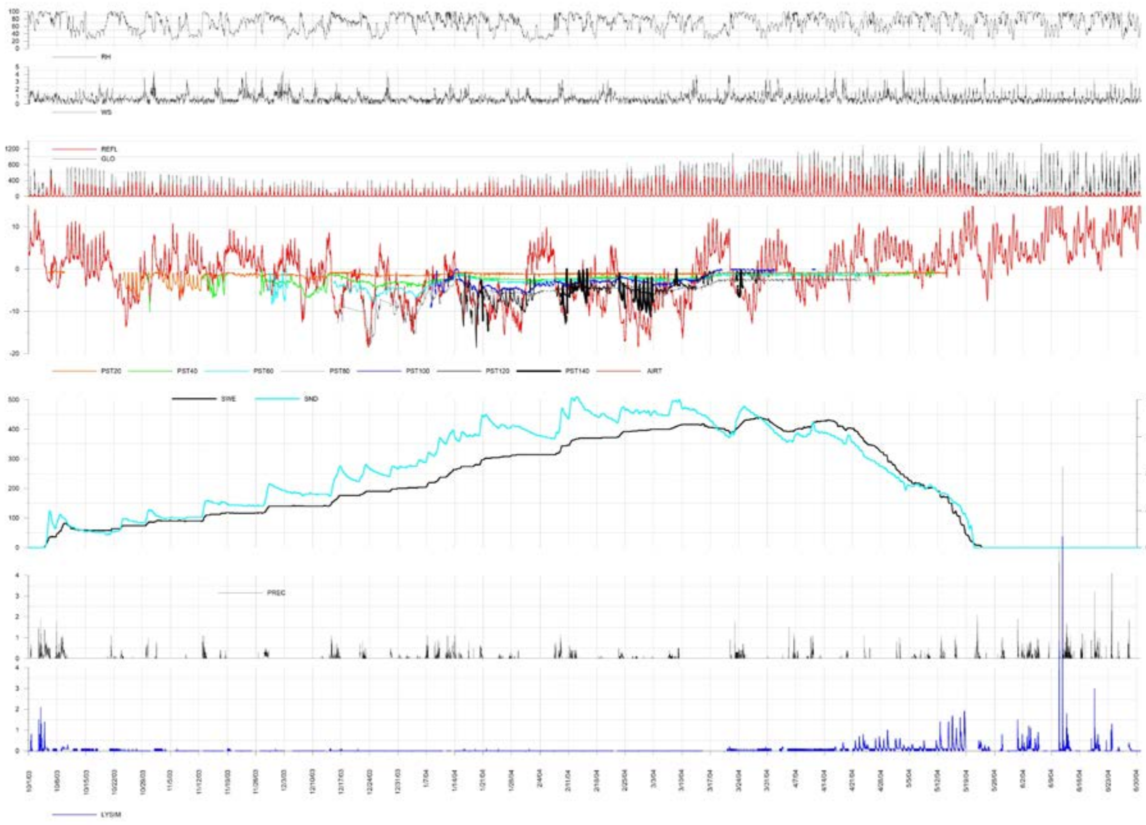

Figure S14. Plots of selected meteorological and snow characteristics in the period October 2003-June 2004.

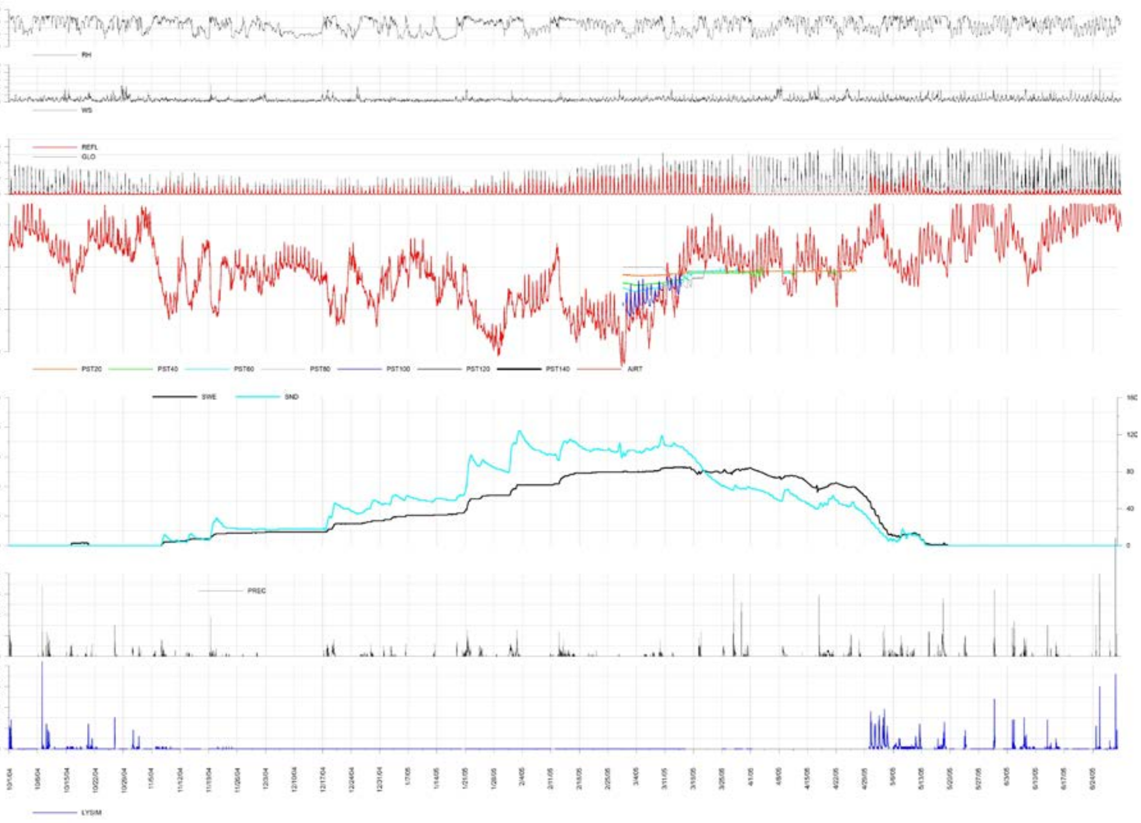

Figure S15. Plots of selected meteorological and snow characteristics in the period October 2004-June 2005.

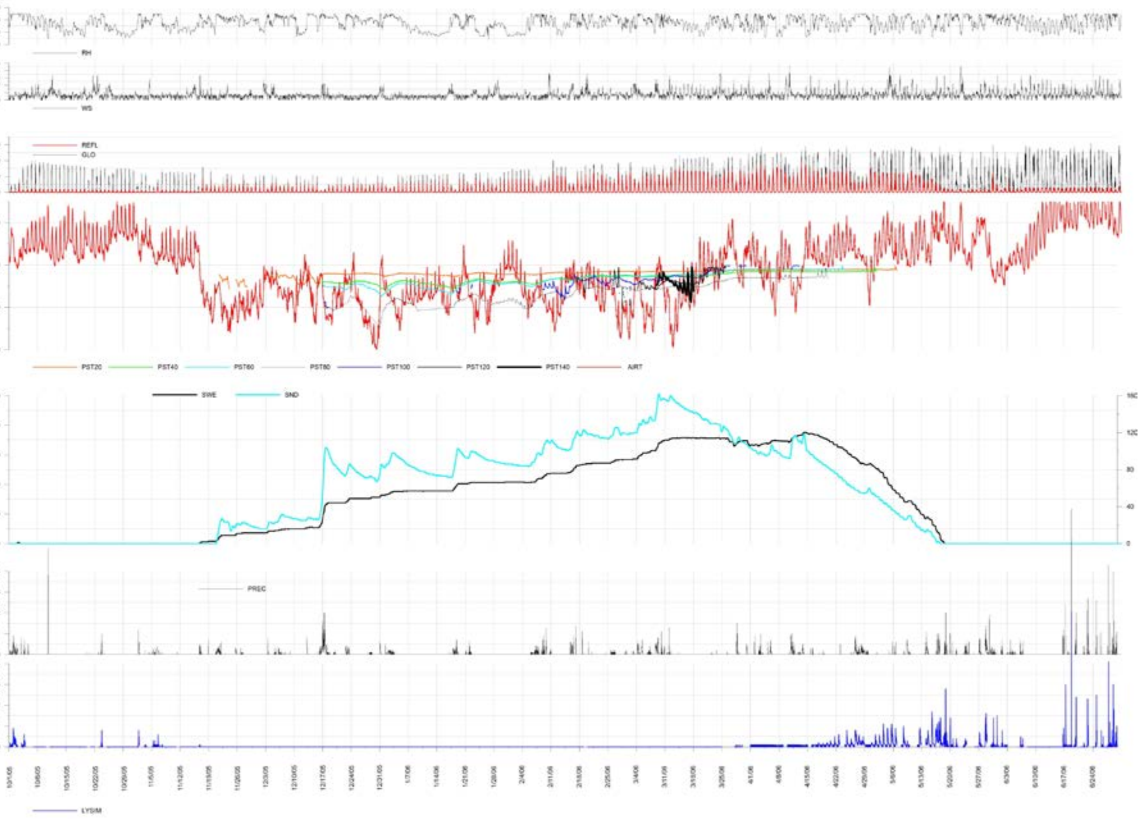

Figure S16. Plots of selected meteorological and snow characteristics in the period October 2005-June 2006.

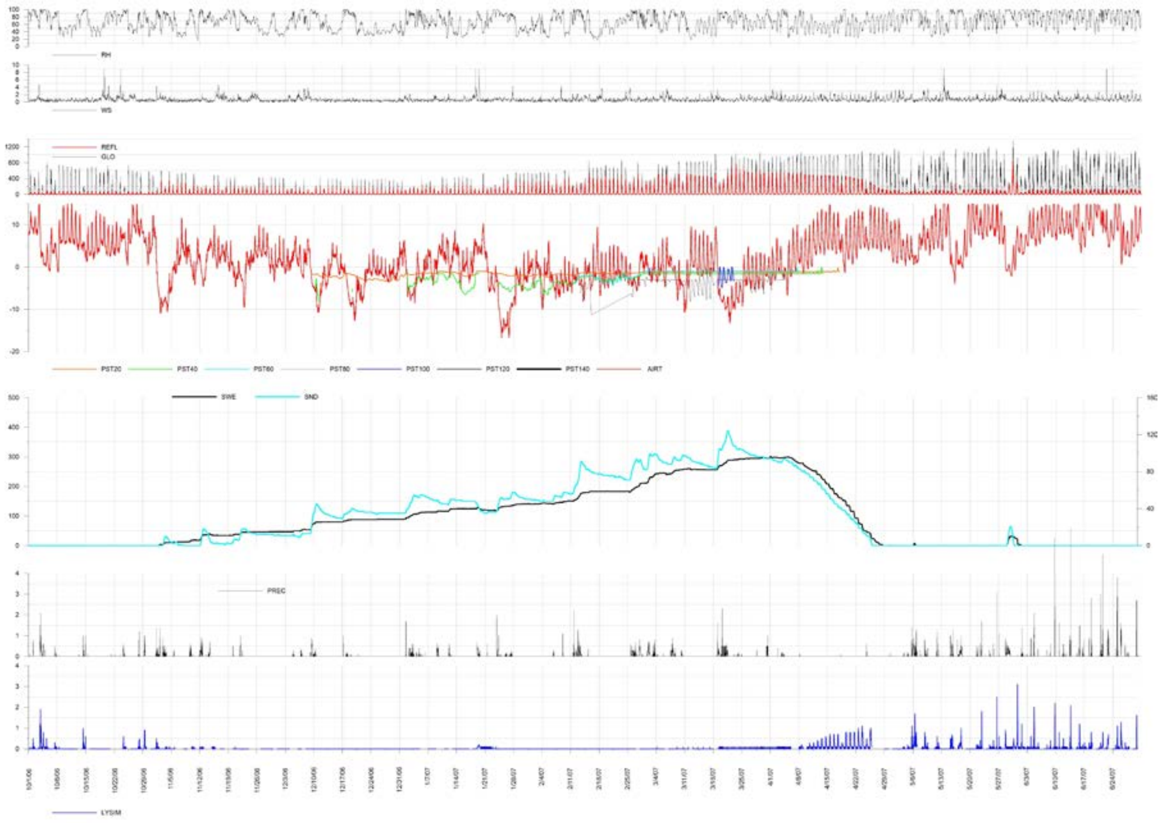

Figure S17. Plots of selected meteorological and snow characteristics in the period October 2006-June 2007.

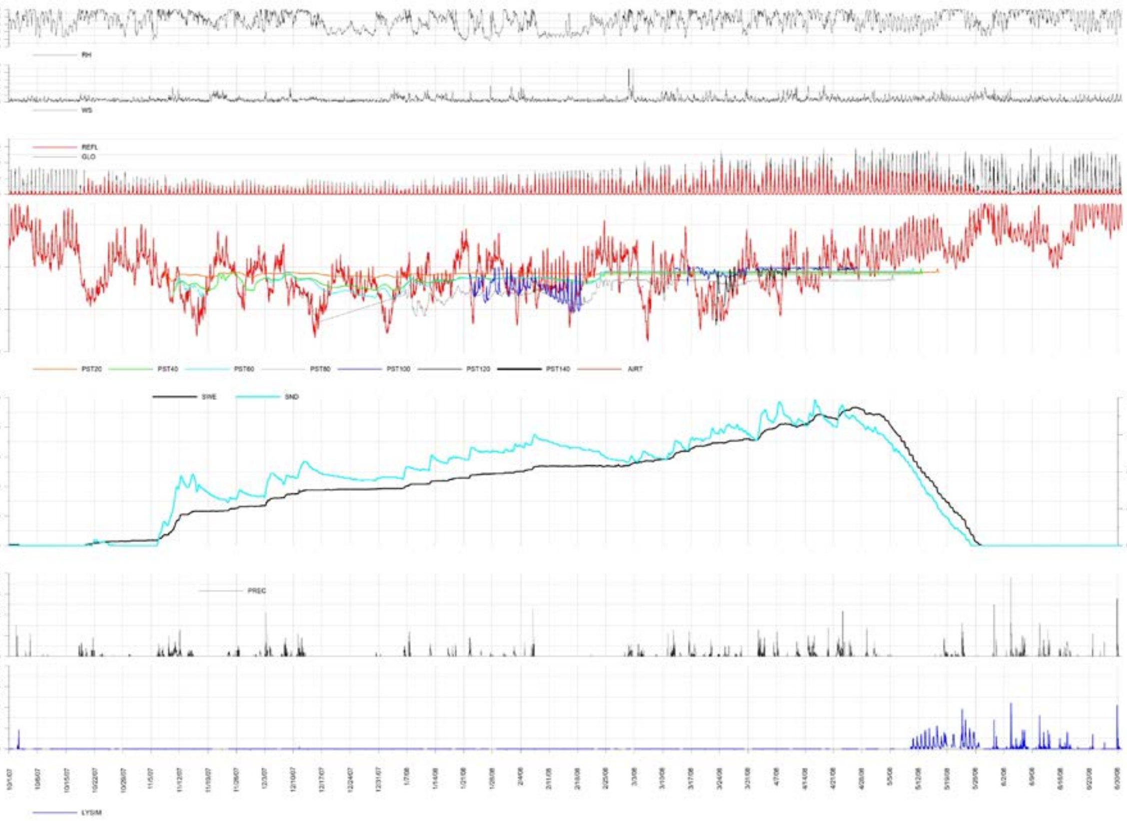

Figure S18. Plots of selected meteorological and snow characteristics in the period October 2007-June 2008.

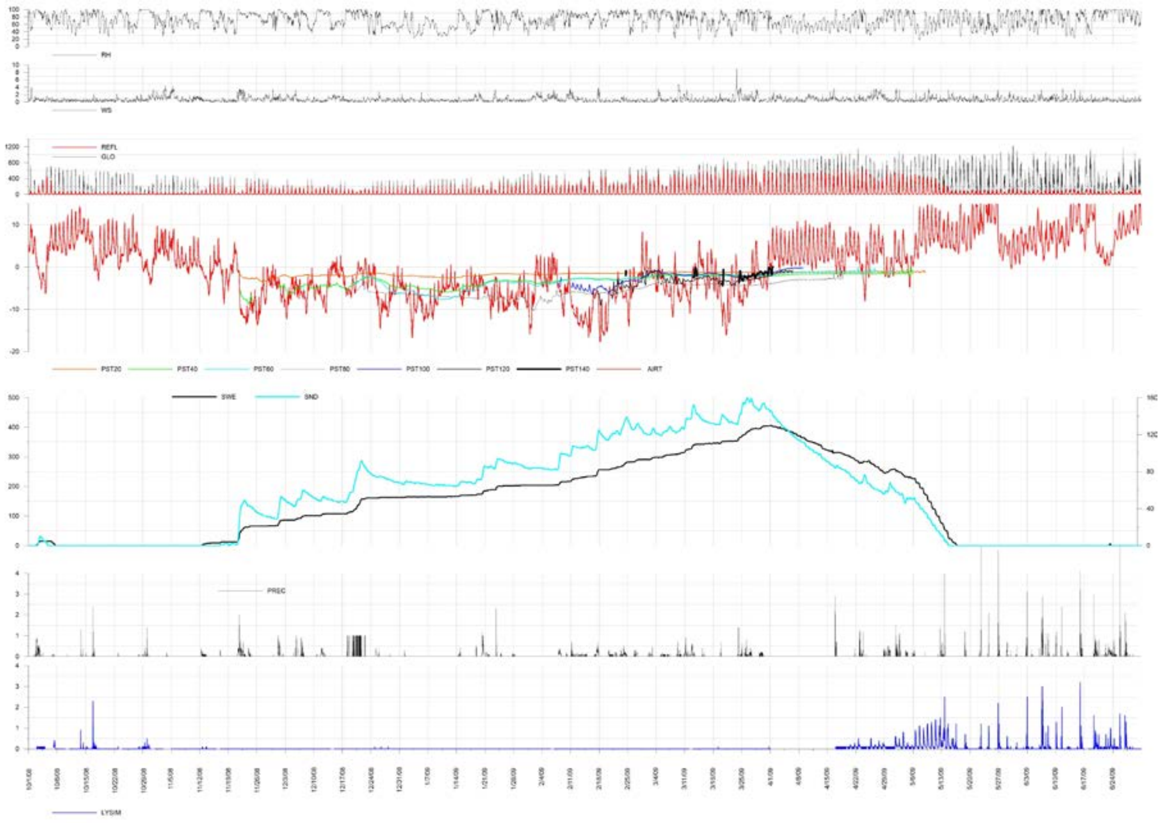

Figure S19. Plots of selected meteorological and snow characteristics in the period October 2008-June 2009.

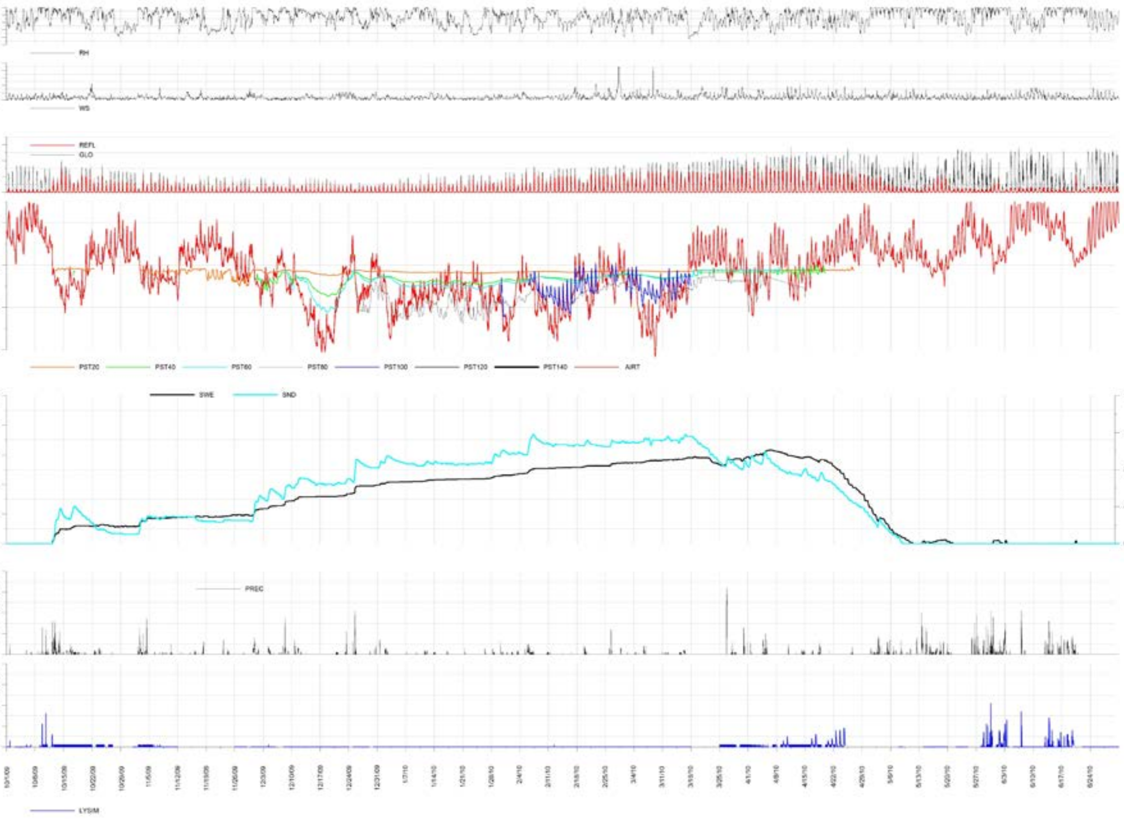

Figure S20. Plots of selected meteorological and snow characteristics in the period October 2009-June 2010.

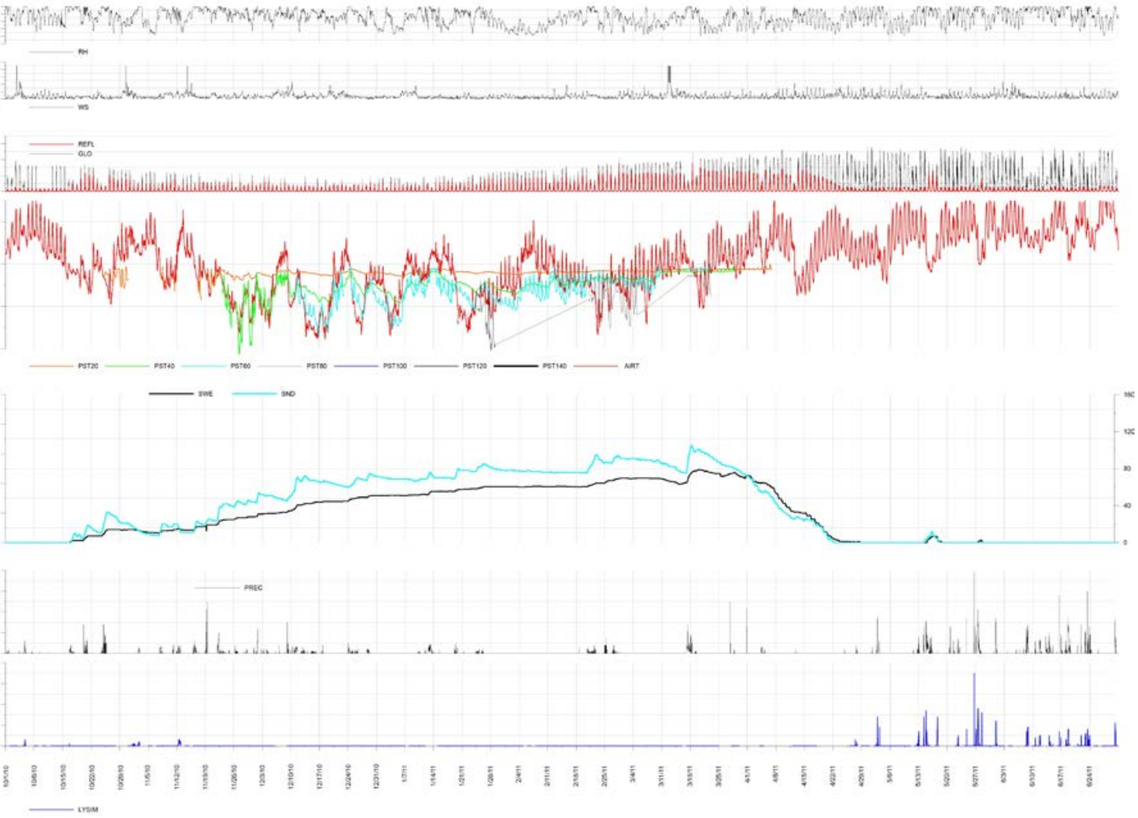

Figure S21. Plots of selected meteorological and snow characteristics in the period October 2010-June 2011.

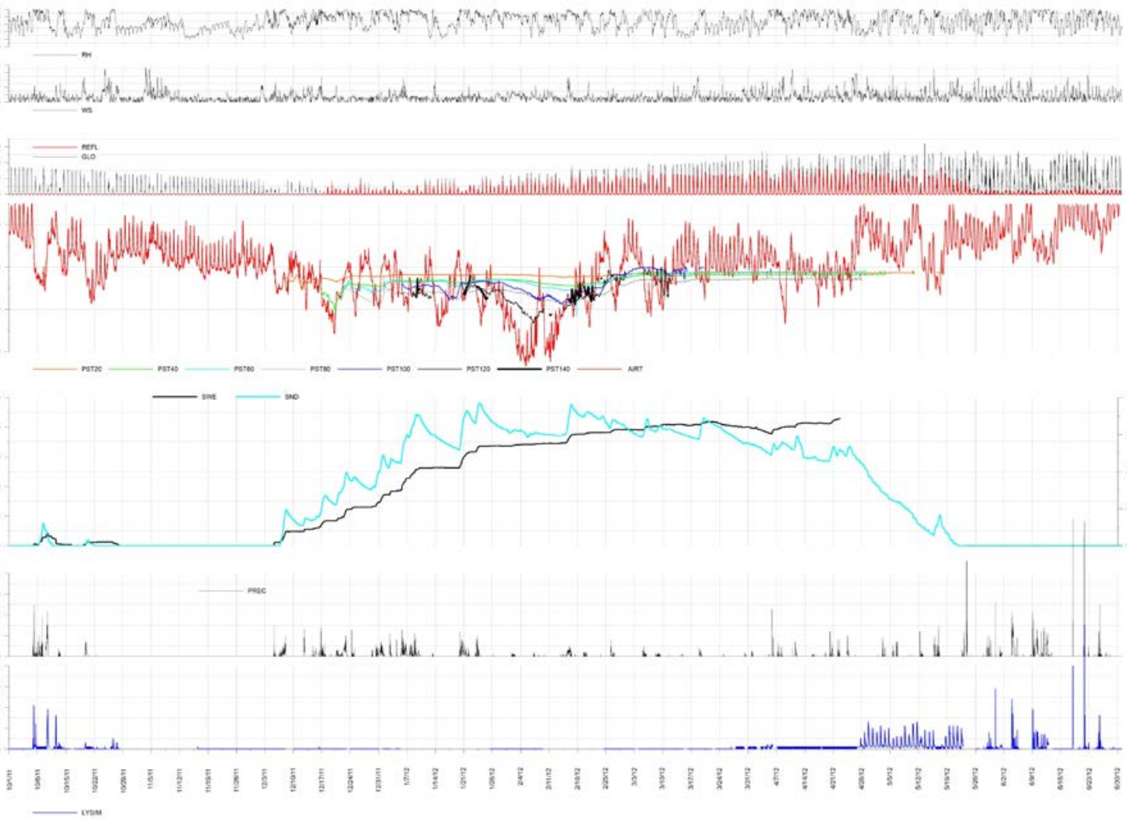

Figure S22 Plots of selected meteorological and snow characteristics in the period October 2011-June 2012.

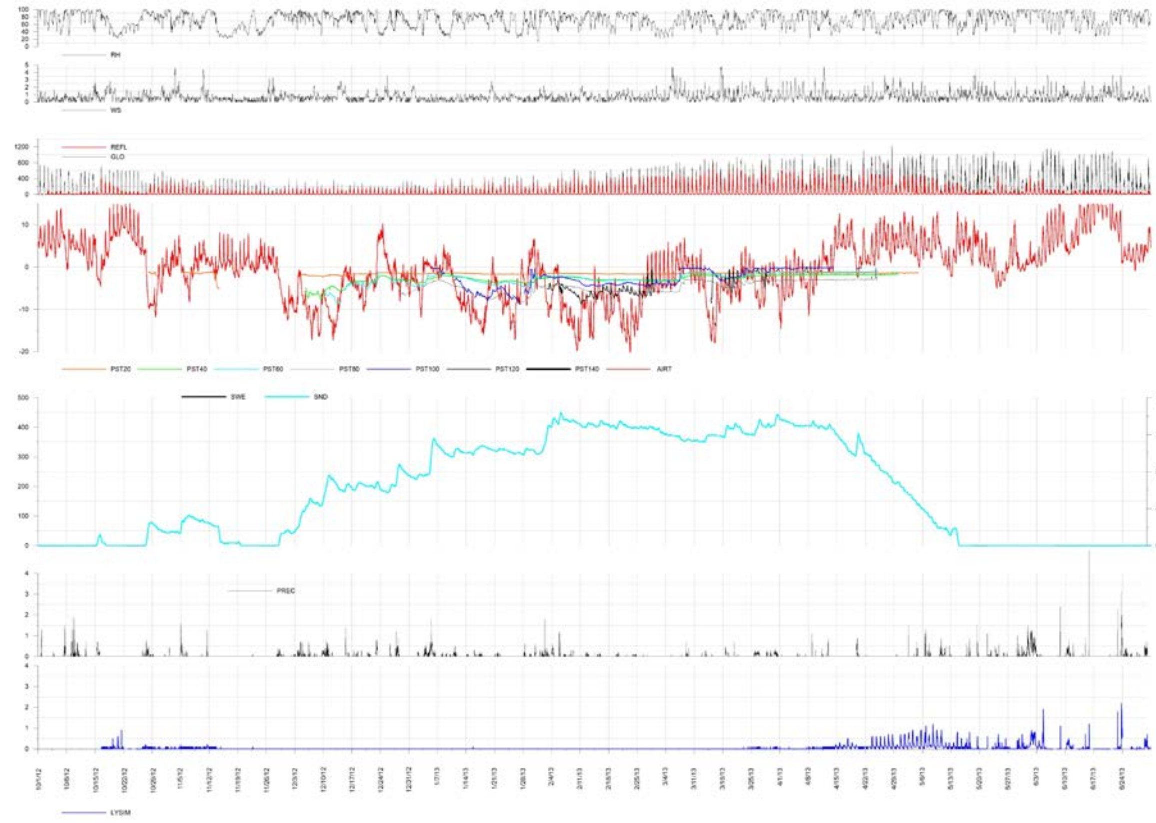

Figure S23. Plots of selected meteorological and snow characteristics in the period October 2012-June 2013.

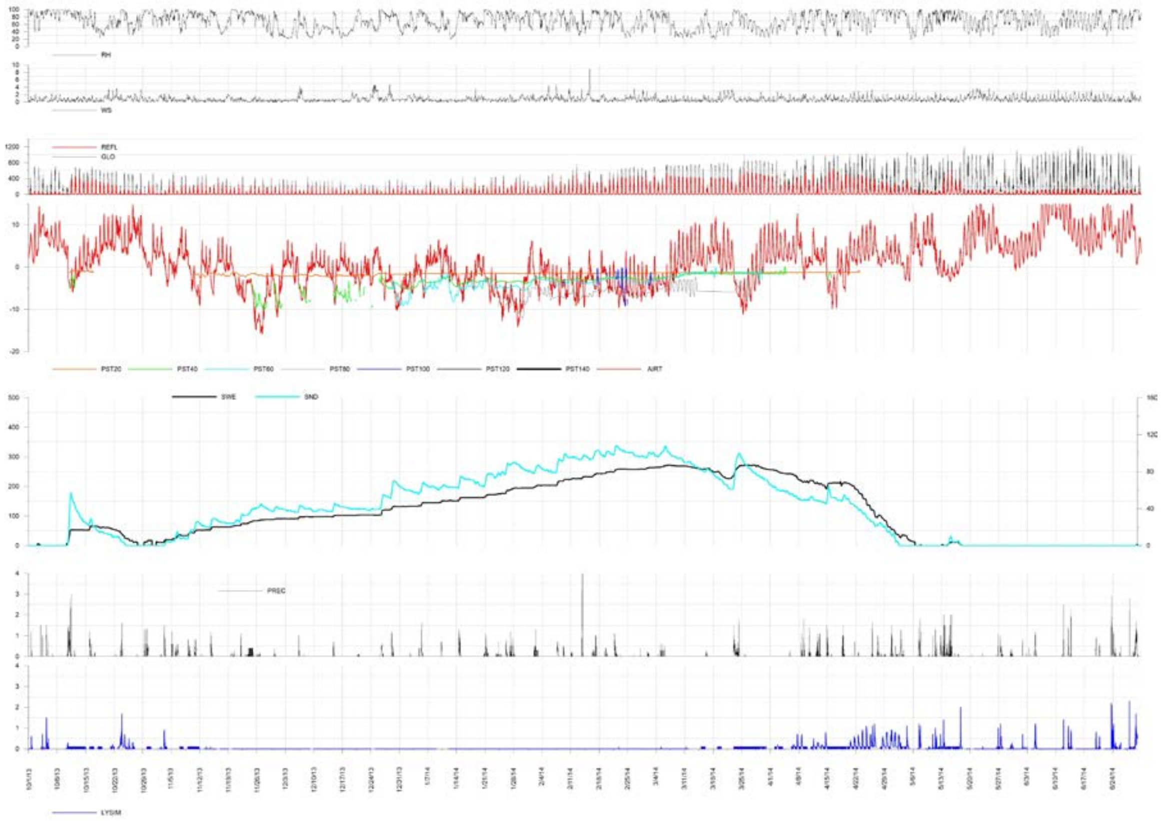

Figure S24. Plots of selected meteorological and snow characteristics in the period October 2013-June 2014.

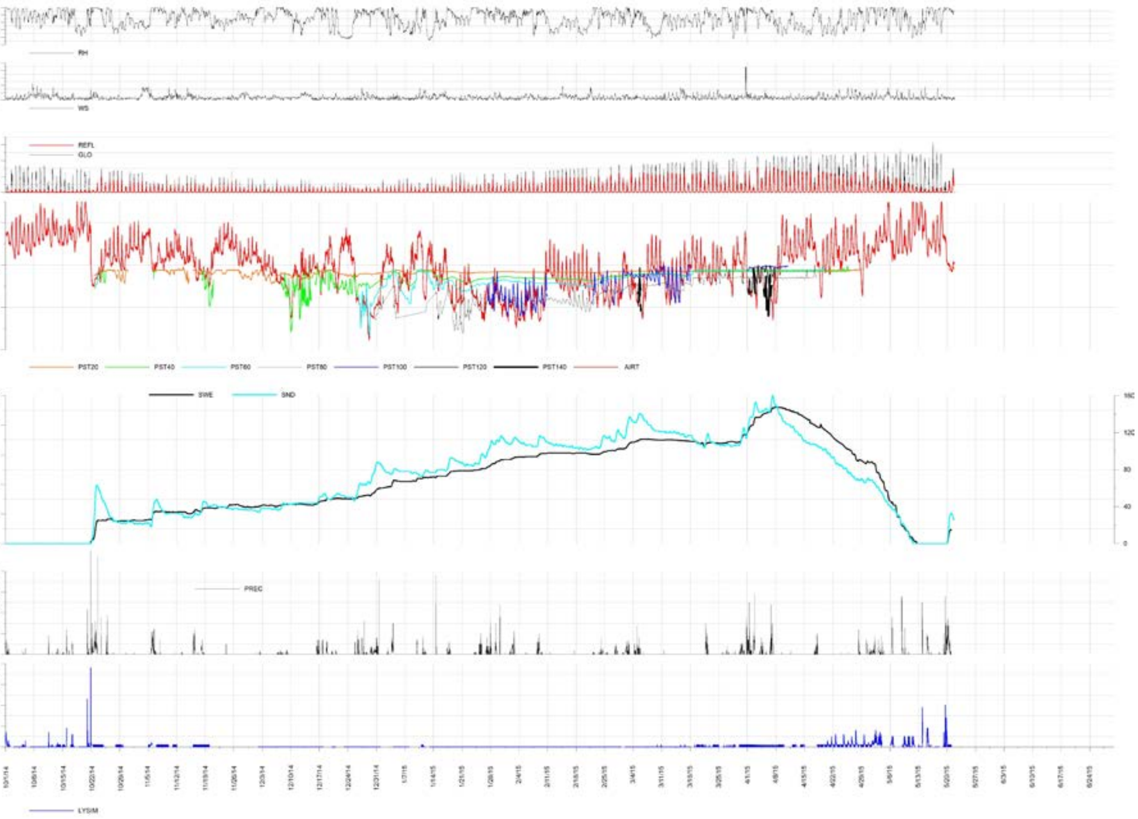

Figure S25. Plots of selected meteorological and snow characteristics in the period October 2014-May 2015.

Data Set S1. The Kühtai data set.
